# Single-item substitutions can substantially reduce the carbon and water scarcity footprints of US diets

**DOI:** 10.1093/ajcn/nqab338

**Published:** 2022-01-13

**Authors:** Donald Rose, Amelia M Willits-Smith, Martin C Heller

**Affiliations:** School of Public Health and Tropical Medicine, Tulane University, New Orleans, LA, USA; School of Public Health and Tropical Medicine, Tulane University, New Orleans, LA, USA; Center for Sustainable Systems, School for Environment and Sustainability, University of Michigan, Ann Arbor, MI, USA

**Keywords:** sustainable diets, carbon footprint, water use, diet quality, diet substitutions, beef, NHANES, 24-h recall

## Abstract

**Background:**

Human food systems substantially affect the environment, but the impacts vary widely by food. Guidance to individuals to reduce their dietary impacts would benefit from easy advice, but little is known about the specific population impacts of simple changes on self-selected diets.

**Objectives:**

The objective was to estimate the potential impact of a single dietary substitution on the carbon and water scarcity footprints of self-selected diets in the United States.

**Methods:**

This cross-sectional modeling study used 24-h dietary recall data from the 2005–2010 waves of the NHANES. Greenhouse gas emissions (GHGE) in the production of foods as well as irrigated water use, characterized by its relative scarcity at production locations, were matched to all foods in the recalls using previously developed databases. Impacts were summed to create carbon and water scarcity footprints for diets (*n*  = 16,800) of adults aged >18 y. Diet quality was assessed using the Healthy Eating Index (HEI). Foods with the highest impact on GHGE and selected additional foods were substituted for calorically equivalent, less impactful items. Footprints were calculated before and after these hypothetical substitutions.

**Results:**

The highest impact foods were all beef items, and 19.8% of individuals consumed them (*n* = 3320). After substitution of these items with poultry or pork, the mean carbon and water scarcity footprints among those with substitutions significantly decreased (*P* < 0.001) by 48.4 ± 0.6% and 29.9 ± 0.4%, respectively. Across the entire sample, these represented mean reductions of 9.6 ± 0.3% and 5.9 ± 0.2%, respectively. The mean HEI after substitutions was 3.6 ± 0.1% higher than before (*P* < 0.001). None of the selected additional foods had population impacts as large as the beef substitutions.

**Conclusions:**

Simple substitutions can be made in individuals’ diets to substantially reduce their carbon and water scarcity footprints without sacrificing dietary quality. Such substitutions may be easier to promote than complex dietary patterns.

## Introduction

Climate change continues to be a defining challenge of our time. Although the historic Paris Climate accords sought to keep temperature levels <1.5 °C above preindustrial levels, current projections indicate a global temperature rise of >3 °C by the end of the century (1). Heat waves, droughts, and heavy rains have increased in frequency and intensity as a result of this warming (2). Human food systems are a key contributor to this problem, accounting for about a third of global greenhouse gas emissions (GHGE) (3). Moreover, ∼70% of global freshwater withdrawals goes to agricultural production (4). Ultimately, consumer demand drives this production, and notable differences in both GHGE and water consumption intensities exist between food types, so dietary guidance is relevant for the environment, not just for health.

The importance of dietary guidance for sustainability has been recognized by nutrition professional organizations (5, 6), government dietary guidance agencies (7–11), and international organizations (12–14). One of the most extensive sets of dietary recommendations developed to meet both health and environmental goals is that of the recent EAT *Lancet* report (13). Unlike many other national recommendations, these guidelines recommend specific foods within broader food groups. For example, within animal protein–rich foods, there are specific recommendations for red meat, poultry, pork, and so on. This is important because of dramatic differences in environmental consequences in the production of these foods. The production of beef creates 8–10 times the GHGE as does the production of chicken and ∼20 times that of nuts, seeds, or legumes (15, 16).

Individuals might like to reduce their environmental footprint, but dietary change is difficult. Not only are diets complex, but habits are hard to break. Moreover, many motivated consumers are bewildered by the choices they face. One strategy to promote change is with simple steps that are easy to understand and implement. Self-efficacy, or the belief in one's ability to exercise control, is often seen as a prerequisite for behavioral change (17, 18). Starting from an existing diet and changing just one aspect of it is easier for consumers than changing to an altogether new diet, because there is less cognitive work involved in understanding and remembering a simple change than there is for a complete diet. It can also require less “willpower,” if the change allows for consumption of a basically equivalent meal.

Although diet change simulations to promote sustainability have been examined previously, most of these have been in the context of adopting complex dietary patterns, such as adopting healthy dietary guidelines, becoming a vegetarian, or shifting to a new sustainable dietary pattern (19–21). We are unaware of any studies that have sought to identify the impacts of a single dietary substitution in the self-selected diets of a nationally representative sample. Here we conduct such a study to examine the potential impacts of single dietary substitutions on the carbon and water scarcity footprints in the United States.

## Methods

### Study sample

Our sample for this study came from NHANES, a nationally representative survey of the civilian, noninstitutionalized population in the United States. NHANES is conducted continuously and is based on a multistage probability design with data released in 2-y waves. Our study used the 3 waves from 2005 to 2010 to match with available data on commodity recipes needed for our environmental impacts (see environmental impact section below). All adults 18 y and older (*N* = 16,800) who completed an acceptable 24-h dietary recall, as defined below under dietary data, were included in our sample.

### Dietary data

Dietary information on NHANES respondents was collected in a Mobile Examination Center by trained interviewers that used an automated multiple pass method for the 24-h dietary recall (22). Based on established survey protocols, diets are considered “acceptable” and included in NHANES for future data analyses if they meet minimum criteria for completeness and contain reliable data. Completeness requires that respondents finish the first 4 steps of the multiple pass method and that there are no missing or unidentified foods. Reliability of the recall is based in part on postrecall interviewer comments, for example, on whether the respondent was uncooperative or could not participate because of memory loss. Additional details about the NHANES diet method, including acceptability of recalls, have been published previously (22–24).

Food items and dishes reported by NHANES respondents are recorded in the recall instrument and can include a substantial amount of detail. There were >6000 foods reported in the 2005–2010 waves of NHANES. These foods are linked through identifying codes to databases on their nutrient content and their equivalent weight in nutritional food groups. Specifically, NHANES food codes are linked to the Food Patterns Equivalent Database (FPED), which allows assigning the ingredients of an NHANES food into groups, such as vegetables, whole grains, and plant protein–rich foods. We used this correspondence and algorithms developed by the National Cancer Institute to calculate a Healthy Eating Index (HEI) score for each individual in the sample (25). The HEI is a previously validated global indicator of diet quality, developed by the USDA and updated with the NIH, designed to provide a metric that corresponds to the recommendations offered in the Dietary Guidelines for Americans (26, 27). It is a 100-point index based on the scores of 12 components: whole fruit; total fruit, including juices; greens and beans; total vegetables; grains; dairy; seafood and plant protein foods; total protein foods; ratio of unsaturated to saturated fatty acids; refined grains; sodium; and empty calories (26). Although all foods contain protein, the term *total protein foods* in the HEI refers to protein-rich foods, such as meats, poultry, fish, eggs, legumes, nuts, and seeds. Seafood and plant protein foods are grouped together in the HEI for scoring purposes because they are both underconsumed in the US population. Calculation of the score also requires linkage of the NHANES nutrient intake data files, which provide the data on sodium and fatty acid intakes. As recommended, the HEI-2010 was used because our data are from 2005 to 2010. See additional information about the HEI in **Supplementary Table 1**.

### Environmental impact data

We considered 2 main environmental impacts from the foods produced for the above diets: GHGE and irrigation water use characterized by scarcity at the source of production. We refer to these respective impacts as the carbon footprint and the water scarcity footprint.

Environmental impacts of food production are typically dominated by agricultural activities and therefore most commonly assessed for commodities, such as wheat, tomato, milk, or pork. However, foods reported by NHANES respondents are often mixed dishes, such as pizza or pasta. To make this bridge between environmental impacts of commodities and dietary intakes, we used 2 different databases. We started with the Food Commodity Intake Database (FCID), which was produced by the US Environmental Protection Agency to translate foods reported in NHANES into a set of recipes with commodities as ingredients (28). FCID is available for 2005–2010 but has not been updated since then, which is why we used these same years of NHANES data for our study. Then we created the database of Food Impacts on the Environment for Linking to Diets (dataFIELD) that summarizes the environmental impact of each of the FCID commodities. To do this, we reviewed the environmental literature for all food life cycle assessment studies between 2005 and 2016, including studies that were either peer-reviewed journal articles or thoroughly documented reports from governmental or nongovernmental organizations. From these studies, we recorded GHGE in kilograms of carbon dioxide equivalents per kilogram of food produced (kg CO_2_-eq/kg) and assigned them to 332 FCID commodities. Environmental impacts from these commodities were then linked to NHANES foods using the recipe file from FCID. See Heller et al. (15) and Rose et al. (29) for additional details of these methods.

We also sought to address the food production impacts on water availability. We developed an analogous database, dataFIELD_water_, which contains an environmental impact metric, “water scarcity intensity,” that assesses the irrigated water used to produce a food, characterized by the relative scarcity of water where it is produced, reported as liter-equivalents per kilogram of food produced (liter-eq/kg). We calculated this for each of the 332 commodities. Production methods vary between farms and across climatic regions, and the irrigation required to yield a kilogram of food varies, as do the associated emissions of carbon dioxide, methane, and nitrous oxide. Whereas fixed factors independent of geography are used to convert these greenhouse gases into global warming potential, our method of converting water use to a metric that addresses potential impacts on humans and ecosystems is dependent on local water scarcity and therefore varies geographically. For example, using the same amount of irrigated water to grow vegetables in a drought-prone place such as California has more environmental impact than in water-rich Louisiana. To address differing production processes as well as differing impacts of resource use, we multiplied geo-specific, watershed-level irrigation inputs by an available water remaining (AWARE) characterization factor. AWARE is a standardized method for assessing the impacts of water use (30) based on the difference between human and ecosystem water demand and the availability of surface and groundwater in a given watershed. See Heller et al. (31) for additional details about this approach.

NHANES does not collect data on the geographic sources of the foods consumed. Thus, we aggregated water impacts by crop to the national level, but we weighted this by the relative production levels in each watershed, which gives a more representative national average than a simple mean. Similar to the process for attributing GHGE, impacts of food commodities were linked to NHANES foods using the FCID recipe files.

Once environmental impacts were linked to each NHANES food, we summed the impacts for all foods consumed by each individual on the recall day to get the overall impact for each individual's diet. These values were scaled by the energy content of the diet to report each impact per 2000 kcal.

### Dietary substitutions

In developing dietary substitutions, we sought to maximize the reduction in diet carbon footprints through simple changes that could be easily remembered and might be readily taken up by consumers. First, we chose target foods for substitution based on the greatest potential impact on climate. All NHANES foods consumed were ranked on the GHGE associated with production. The ranking of each food was done in 2 ways, by the total emissions to produce this food for the total amount consumed by the overall sample, expressed in kg CO_2_-eq, and by the relative intensity of emissions, expressed in kg CO_2_-eq per 100 g of food, as consumed. Both values accounted for losses at the retail and consumer levels [methods detailed in Heller et al. (15)]. Both rankings are important. The first indicates which foods could influence population-level impacts the most, whereas the second gives the individual foods with the highest impacts, even if not commonly consumed in the sample. This could be useful for individuals who consume these high-impact foods and wish to make a difference in their own footprints. **Supplementary Tables 2** and**3** list the top 100 foods ranked by overall impact and by intensity of impact, respectively.

Then, we selected all foods that were in the top 100 of both rankings. This yielded a list of 25 foods, which we ranked by overall impact. We chose to target the top 10 foods on this list for substitutions (**Supplementary Table 4**). All of these foods were either cuts of beef or ground beef items. NHANES uses very detailed definitions for its foods to better assess the nutrient content of respondent food consumption. For example, ground beef that is 90–94% lean is a different food than ground beef that is 85–89% lean. There is no difference in environmental impact between cuts of beef in our assessment, but they show up in different rankings in our Supplementary Table 4 because they were consumed in different amounts by the NHANES respondents. To keep our substitution strategy simple for potentially interested consumers, we decided to substitute for all *cuts of beef or ground beef items*, regardless of whether they were in the top 10 of this list.

Finally, we examined the impacts of a single hypothetical substitution for any individual who reported consuming one of these high-impact foods. An alternative NHANES foods was used to substitute for one of these high-impact foods. Our approach was to maintain caloric and culinary equivalence, while substituting with a lower impact food. By culinary equivalence, we mean roughly similar cooking, tasting, and eating properties. In most cases, we simply substituted a poultry item for a beef item. For example, for “beef, not specified as to cut, cooked, not specified as to fat eaten,” we substituted “chicken, not specified as to part and cooking method, not specified as to skin eaten.” Because ground turkey is more commonly available than ground chicken, we used that to substitute for ground beef. We also used some pork products as substitutions, for example, pork spareribs for beef short ribs. See **Supplementary Table 5** for a complete list of the 64 substitutions that were implemented.

Because all of the substitutions were NHANES foods that were linked to nutrient content and FPED food groups and because we had previously assigned environmental impacts to them, it was straightforward to calculate the environmental impacts and HEI for the new diets after substitutions had been made.

Although the main substitution of interest was that in which a single change would have the maximum impact on climate, we also examined selected additional substitutions to provide an illustration of the range of such substitutions and their potential impacts. These selections were from each of the broad food groups in the Dietary Guidelines for Americans, including fruit, vegetables, grains, dairy, and protein-rich foods. The specific choices were based on the authors’ judgments as to which would most influence the dietary carbon footprint or the water scarcity footprint. These judgments were informed by our previous research with these data (15, 31) and by the top 100 lists we created for this article to rank NHANES foods on their environmental impacts (see **Supplementary Tables**2–4 and**6–8**).

In all scenarios, if an individual consumed the target food more than once per day, only 1 substitution was made in our analyses, that with the highest GHGE value.

### Characteristics of the sample

The characteristics of our sample are described with various socioeconomic and other variables, including sex, age group (18–29, 30–49, 50–65, 66 y and older), self-identified race and ethnicity (Hispanic, non-Hispanic white, non-Hispanic black, other), education (less than high school, high school graduate or equivalent, some college, college graduate or higher), and income. The latter was expressed as a ratio of family income to the poverty threshold and categorized into 4 groups (<1, 1–1.99, 2–4.99, 5+). Diets of individuals were also ranked by energy-adjusted GHGE (kg CO_2_-eq/2000 kcal) and divided into quintiles, the fifth being the diets with the highest impact.

### Ethics

This research project, based on publicly available and deidentified secondary data, meets the federal criteria for exemption as determined by the Tulane University Human Subjects Protection Program.

### Statistical analysis

NHANES uses a complex multistage probability sampling design that allows them to efficiently gather a nationally representative sample as well as accurately provide estimates on underrepresented groups. To analyze data from their public use files, NHANES provides individual sampling weights and complex design parameters, which identify the primary sampling unit and the stratum of each observation. As recommended, we used these design parameters along with the day 1 dietary sample weights for estimating all results presented here and in the supplementary tables. Chi-square statistics were used for tests of associations between demographic variables and whether or not the individual had a dietary recall that included a substituted food. Paired *t* tests were conducted for differences between a respondent's original diet and his or her new diet after the substitution for both environmental impacts as well as for the HEI. An α level of 0.05 was used to determine statistical significance. All calculations and analyses were conducted in Stata/SE 13.1 (StataCorp).

**TABLE 1:** Demographic characteristics of the study sample and those who received substitutions, NHANES 2005–2010

Sample characteristic	Overall sample (*N* = 16,800), %	Had substitutions^1^ (*n* = 3320), %	*P* value^2^
Sex	—	—	<0.001
Women	52.1	45.2	
Men	47.9	54.8	
Age, y	—	—	0.0079
18–29	22.1	23.1	
30–49	36.9	39.0	
50–65	25.5	25.3	
66+	15.5	12.5	
Race-ethnicity	—	—	0.1488
Hispanic	12.7	13.9	
Non-Hispanic white	70.1	70.1	
Non-Hispanic black	11.6	11.0	
Other	5.7	5.1	
Education	—	—	0.0257
Less than high school	19.2	20.1	
High school graduate/GED	25.0	27.3	
Some college	30.6	29.7	
College graduate or higher	25.2	22.9	
Income to poverty ratio^3^	—	—	0.8463
Missing income	6.2	6.0	
<1	13.2	13.4	
1 to <2	19.1	18.5	
2 to <5	37.0	37.1	
5+	24.4	25.1	
Quintile of dietary GHGE^4^	—	—	<0.001
First	20.0	0.8	
Second	20.0	2.5	
Third	20.0	9.6	
Fourth	20.0	31.9	
Fifth	20.0	54.3	

1 One single-item substitution was made for any individual who consumed one of the high-impact foods, either ground beef or a cut of beef, on their 24-h recall day. See Supplementary Table 5 for the high-impact foods and their substitutions.

2 *P* value determined from a χ^2^ test.

3 Income to poverty ratio is the ratio of family income to the poverty guideline for each family based on its size, state of residence, and the year of observation.

4 GHGE is the greenhouse gas emissions associated with the diets of individuals and is measured in kilograms of carbon dioxide equivalents (kg CO_2_-eq/2000 kcal). Diets were ranked on this measure and divided into quintiles, the fifth being the diets with the highest impact.

## Results

The demographic and socioeconomic characteristics of our sample are presented in the first data column of **Table 1**. Adults, 30 to 49 y of age, made up 37% of our sample, the largest of the 4 age groups, whereas females made up 52% of our sample. Non-Hispanic whites (70%) were the largest racial-ethnic group, followed by Hispanics (13%) and non-Hispanic blacks (12%). A majority of the sample had some college education or higher. Those with family incomes below the poverty threshold comprised 13% of the sample, while about a quarter of the sample had a family income 5 times the poverty threshold or more.

The second data column of Table 1 reports the characteristics of those individuals for whom we substituted a food on the recall day. There were 3320 of these individuals, 19.8% of the sample. Sex, age, and education were all significantly associated with those who had substituted foods on the recall day. Specifically, men were more likely than women to have a substituted food, and older adults (66+ y) were less likely than younger adults to have a substitution. Those with a college education, particularly college graduates, were also less likely to have a substitution. Neither race-ethnicity nor family income was significantly related to those who had substitutions. Most of the substitutions (86%) were made in diets that were ranked in the top two quintiles of dietary GHGE. Of those that had substitutions, most (88%) only consumed the high-impact food once that day, but 11% consumed it twice and 1% consumed it three or more times on the recall day (results not shown).

An example 24-h recall for one individual (#54,886) from our sample is presented in **Table 2** with each food reported by the meal in which it was eaten along with the amount, calorie value, and GHGE of that food. The GHGE for ground beef (item 10), 3.97 kg CO_2_-eq, was the highest in this diet, much higher than any other food eaten on that day. All other foods had a GHGE <0.5 kg CO_2_-eq, except for pork roast (item 19) at 0.67 kg CO_2_-eq. Overall, foods and drinks in this diet summed to 2004 kcal and 6.68 kg CO_2_-eq. Ground turkey was used as the substituted food, and the GHGE to produce an equal calorie amount of this food was 0.41 kg CO_2_-eq. After substituting in ground turkey instead of the ground beef, the total GHGE for the new diet would be 3.12 kg CO_2_-eq, 53% lower than the original.

**TABLE 2:** Weight consumed, kilocalories, and greenhouse gas emissions of the items listed in the diet recall for 1 individual (#54,886) before and after a single-item substitution^1^

Item	Meal^2^	Food description	Grams	kcal	GHGE^3^
1	M1	Granola, low fat, Kellogg's	73.5	284	0.060
2	M1	Milk, cow, fluid, skim or nonfat	122.5	42	0.168
3	M1	Cranberries, dried	27.5	85	0.050
4	M1	Banana, raw	118.0	105	0.058
5	M1	Pineapple juice	203.1	108	0.440
6	M1	Coffee, espresso	59.2	1	0.011
7	M1	Sugar, white, granulated or lump	4.2	16	0.004
8	M1	Water, tap	118.5	0	0.000
9	S1	Pecans	7.5	52	0.019
10	M2	Ground beef, 95% or more lean, cooked	94.3	153	3.969
11	M2	Bread, whole wheat, NS as to 100%	26.0	68	0.012
12	M2	Wine, table, red	102.9	87	0.122
13	M2	Broccoli, cooked, fat added in cooking	241.5	142	0.242
14	M2	Applesauce, stewed apples, unsweetened	366.0	154	0.121
15	M2	Water, tap	118.5	0	0.000
16	D1	Coffee, espresso	59.2	1	0.011
17	D1	Sugar, white, granulated or lump	4.2	16	0.004
18	D2	Water, tap	237.0	0	0.000
19	M3	Pork roast, NS as to cut, cooked, lean only	84.0	175	0.668
20	M3	Bread, whole wheat, NS as to 100%	18.0	47	0.008
21	M3	Olive oil	6.8	60	0.030
22	M3	Olive oil	13.5	119	0.061
23	M3	Tomatoes, raw	85.0	15	0.049
24	M3	Lettuce, arugula, raw	10.0	3	0.004
25	M3	Onions, mature, raw	20.0	8	0.009
26	M3	Wine, table, red	102.9	87	0.122
27	M3	Cantaloupe (muskmelon), raw	516.8	176	0.442
28	M3	Water, tap	118.5	0	0.000
29	D3	Water, tap	118.5	0	0.000
		Total kcal and GHGE in original diet		2004	6.684
					
		Substitution for item 10			
10S	M2	Turkey, ground	104.6	153	0.409
		Total kcal and GHGE in substitution diet		2004	3.124

1 NS, not specified.

2 Meals (M), snacks (S), or drinks (D), identified here numerically in the order during the day they were eaten or drunk.

3 GHGE refers to greenhouse gas emissions from the production of each food and is measured in kilograms of carbon dioxide equivalents (kg CO_2_-eq).

For diets with substitutions in our sample, the new diets averaged 3.73 kg CO_2_-eq per 2000 kcal less than the original diets (*P*  < 0.001), a mean reduction of 48.4% (**Table 3**). The water scarcity footprint for new diets averaged 972 liter-eq per 2000 kcal less than the original diets (*P* < 0.001), which was a mean reduction of 29.9%.

**TABLE 3:** Mean changes in environmental impacts after a single-item substitution among those with substitutions and for the entire sample^1^

Environmental impact	Before substitution, mean ± SE	After substitution, mean ± SE	Absolute difference, mean ± SE	Percentage difference, mean ± SE	*P* value^2^
For those with substitutions^1^ (*n* = 3320)					
Carbon footprint, kg CO_2_-eq 2000 kcal^–1^	7.23 ± 0.07	3.51 ± 0.04	–3.73 ± 0.07	–48.4 ± 0.6	<0.001
Water scarcity footprint, liter-eq 2000 kcal^–1^	3268 ± 45	2297 ± 38	–972 ± 18	–29.9 ± 0.4	<0.001
For the entire sample^3^ (*n* = 16,800)					
Carbon footprint, kg CO_2_-eq 2000 kcal^–1^	4.42 ± 0.03	3.68 ± 0.03	–0.74 ± 0.03	–9.6 ± 0.3	<0.001
Water scarcity footprint, liter-eq 2000 kcal^–1^	2542 ± 27	2349 ± 26	–192 ± 7	–5.9 ± 0.2	<0.001

1 One single-item substitution was made for any individual that consumed one of the high-impact foods, either ground beef or a cut of beef, on his or her 24-h recall day. If the individual consumed the food more than once on that day, only 1 instance, the one with the highest carbon footprint, was substituted. See Supplementary Table 5 for the high-impact foods and their substitutions. kg CO_2_-eq, kilograms of carbon dioxide equivalents; liter-eq, liter-equivalent.

2 *P* value determined from a paired *t* test.

3 These results describe mean environmental impacts assessed for the entire sample, even though substitutions were only made on a subsample of 3320 individuals.

Although the above-described substitutions were made on just 19.8% of the diets, the potential impact for the entire sample can be obtained by averaging the impacts of these changes with the 80.2% of diets in which there were no substitutions and therefore had zero changes in environmental impacts (Table 3, bottom half). When considered in this way, the above substitutions would result in an overall decrease in GHGE of 9.6% and a 5.9% drop in the water scarcity footprint.

The HEI score for new diets averaged about 1.5 points (or 3.6%) higher than for the original diets (*P* < 0.001; **Table 4**). This was largely driven by improvements in the subcomponents on fatty acids and empty calories, in which foods with lower concentrations of saturated fats would improve the scores. The sodium content of substituted foods was higher than the original items, accounting for a decrease in the average score of this subcomponent.

**TABLE 4:** Mean changes in the Healthy Eating Index (HEI) component and overall scores after a single-item substitution among those with substitutions^1^

HEI component score	Before substitution	After substitution	Absolute difference^2^	Percentage difference^2^	*P* value^3^
For those with substitutions (*n* = 3320)^4^					
Total fruit	1.9 ± 0.1	1.9 ± 0.1	—	—	—
Whole fruit	1.8 ± 0.1	1.8 ± 0.1	—	—	—
Total vegetables	3.0 ± 0.0	3.0 ± 0.0	0.01 ± 0.00	0.3 ± 0.1	<0.001
Greens and beans	1.2 ± 0.1	1.2 ± 0.1	0.02 ± 0.00	4.2 ± 1.2	<0.001
Whole grains	1.7 ± 0.1	1.7 ± 0.1	—	—	—
Dairy	5.0 ± 0.1	5.0 ± 0.1	—	—	—
Total protein foods^5^	4.6 ± 0.0	4.7 ± 0.0	0.08 ± 0.01	2.5 ± 0.2	<0.001
Seafood and plant proteins^6^	1.6 ± 0.1	1.5 ± 0.1	–0.02 ± 0.01	–1.2 ± 0.3	<0.001
Fatty acids	3.8 ± 0.1	4.9 ± 0.1	1.11 ± 0.03	116.4 ± 13.6	<0.001
Refined grains^7^	6.8 ± 0.1	6.8 ± 0.1	—	—	—
Sodium^7^	5.1 ± 0.1	4.6 ± 0.1	−0.52 ± 0.03	−12.3 ± 0.7	<0.001
Empty calories^7^^,^ ^8^	11.4 ± 0.1	12.2 ± 0.1	0.83 ± 0.03	19.6 ± 3.1	<0.001
Total HEI score	48.1 ± 0.5	49.6 ± 0.5	1.5 ± 0.04	3.6 ± 0.1	<0.001

1 One single-item substitution was made for any individual who consumed one of the high-impact foods, either ground beef or a cut of beef, on his or her 24-h recall day. See Supplementary Table 5 for the high-impact foods and their substitutions. Values are mean ± SE. The Healthy Eating Index (HEI) is an overall index of diet quality based on the Dietary Guidelines for Americans. The 2010 version was used for this analysis (28). See Supplementary Table 1 for additional details about scoring. Component scores with dashes in the 3rd, 4th, and 5th data columns were not affected by the substitutions.

2 Differences in scores between the original and new diets, as well as percentage differences, were calculated for each individual and then averaged across those with substitutions.

3 Determined by paired *t* test.

4 Percentage changes often have a different number than the subsample of 3320 because a number of people started with scores of zero. In the percentage difference column, numbers for greens and beans, seafood and plant proteins, fatty acids, sodium, and empty calories were 3310, 3319, 3126, 3303, and 3272, respectively.

5 “Total protein foods” in the HEI refers to protein-rich foods, such as meats, poultry, fish, eggs, legumes, nuts, and seeds.

6 “Seafood and plant proteins” refers to fish, seafood, legumes, nuts, and seeds. They are grouped together for scoring purposes because they are underconsumed in the US diet.

7 Higher component scores are considered beneficial. Thus, for refined grains, sodium, and empty calories, higher scores indicate diets that contain less of these items.

8 Calories from solid fats, added sugars, and alcohol. For alcohol, intakes ≤13 g/1000 kcal do not influence scoring.

Although beef was our primary substitution of interest because our rankings showed it would have the greatest potential impact on climate, we also examined a number of other potential food substitutions. The environmental impacts of these are shown in **Table 5**. Of these alternate substitutions, the greatest impacts on GHGE came from replacing shrimp with cod (34.1% reduction) and from replacing dairy milk with soymilk (8.1% reduction). The greatest reduction in the water scarcity footprint came from replacing asparagus with peas, resulting in a 48.2% decrease. Substituting peanuts in place of almonds decreased the water scarcity footprint by 30.4%.

**TABLE 5:** Mean absolute and percentage changes in environmental impacts after single-item substitutions for selected additional foods^1^

			Carbon footprint			Water scarcity footprint		
Original food	Substituted food	*n* (%)^2^	Absolute change, mean ± SE, kg CO_2_-eq/2000 kcal	Percentage change, mean ± SE, %	*P* value^3^	Absolute change, mean ± SE, liter-eq/2000 kcal	Percentage change, mean ± SE, %	*P* value^3^
Grapes	Apples	1144 (7.1)	–0.01 ± 0.00	–0.4 ± 0.1	<0.001	–573 ± 20	–16.7 ± 0.5	<0.001
Lemon juice	Orange juice	447 (2.3)	–0.00 ± 0.00	–0.1 ± 0.0	<0.001	–193 ± 25	–6.7 ± 0.6	<0.001
Asparagus	Peas	160 (1.3)	–0.27 ± 0.02	–6.5 ± 0.5	<0.001	–3352 ± 197	–48.2 ± 1.6	<0.001
Broccoli	Brussel sprouts	644 (4.1)	–0.05 ± 0.00	–1.3 ± 0.1	<0.001	–652 ± 31	–18.0 ± 0.6	<0.001
White bread	Whole-wheat bread	6087 (37.6)	–0.00 ± 0.00	–0.0 ± 0.0	0.6934	2 ± 0	0.1 ± 0.0	<0.001
White rice	Bulgur wheat	1617 (8.7)	–0.11 ± 0.00	–3.5 ± 0.1	<0.001	–116 ± 3	–5.5 ± 0.2	<0.001
Dairy milk	Soymilk	6995 (43.1)	–0.28 ± 0.00	–8.1 ± 0.1	<0.001	–99 ± 2	–5.0 ± 0.1	<0.001
Almonds	Peanuts	315 (2.4)	–0.07 ± 0.00	–2.5 ± 0.3	<0.001	–1397 ± 97	–30.4 ± 1.4	<0.001
Shrimp	Cod	480 (2.6)	–2.82 ± 0.25	–34.1 ± 1.3	<0.001	–131 ± 12	–6.1 ± 0.5	<0.001
Beef^4^	Chicken/pork	3320 (19.8)	–3.73 ± 0.07	–48.4 ± 0.6	<0.001	–972 ± 18	–29.9 ± 0.4	<0.001

1 Single-item substitutions were made for any individual who consumed one of the above foods. If the individual consumed the food more than once on the 24-h recall day, only 1 instance, the one with the highest carbon footprint, was substituted. See **Supplementary Table 9** for the specific foods that were substituted. kg CO_2_-eq, kilograms of carbon dioxide equivalents; liter-eq, liter-equivalent.

2 Number and percentage of respondents who consumed this food and for whom the substituted food was tested.

3 *P* value determined from a paired t-test.

4 This is the main substitution from Table 3 that is displayed again here for convenience.

To what extent did these single-item substitutions reduce the environmental impacts of the overall population-wide US diet? Answers to this question are presented in **Table 6**, which take into account the mean reductions shown in Table 5 among those who consumed the substituted foods as well as the percentage of sampled individuals consuming them. The beef substitution reduced the overall impact on GHGE by 9.6%, which was over twice the percentage reduction of the milk substitution (3.5%) and >10 times the reduction of the shrimp substitution (0.9%). On a population-wide basis, the beef substitution also reduced the water scarcity footprint by 5.9%, over twice that of the dairy substitution (2.2% reduction), >4 times the grape substitution (1.2% reduction), and >7 times the almond substitution (0.7%).

**TABLE 6:** Mean changes in environmental impacts across the entire sample after a single-item substitution of selected additional foods among those who consumed them^1^

			Carbon footprint			Water scarcity footprint		
Original food	Substituted food	*n* (%)^2^	Absolute change, mean ± SE, kg CO_2_-eq/2000 kcal	Percentage change, mean ± SE, %	*P* value^3^	Absolute change, mean ± SE, liter-eq/2000 kcal	Percentage change, mean ± SE, %	*P* value^3^
Grapes	Apples	1144 (7.1)	0.00 ± 0.00	0.0 ± 0.0	<0.001	–41 ± 2	–1.2 ± 0.1	<0.001
Lemon juice	Orange juice	447 (2.3)	0.00 ± 0.00	0.0 ± 0.0	<0.001	–4 ± 1	–0.2 ± 0.0	<0.001
Asparagus	Peas	160 (1.3)	0.00 ± 0.00	–0.1 ± 0.0	<0.001	–43 ± 7	–0.6 ± 0.1	<0.001
Broccoli	Brussel sprouts	644 (4.1)	0.00 ± 0.00	–0.1 ± 0.0	<0.001	–27 ± 2	–0.7 ± 0.1	<0.001
White bread	Whole-wheat bread	6087 (37.6)	0.00 ± 0.00	0.0 ± 0.0	0.6932	1 ± 0	0.0 ± 0.0	<0.001
White rice	Bulgur wheat	1617 (8.7)	–0.01 ± 0.00	–0.3 ± 0.0	<0.001	–10 ± 1	–0.5 ± 0.0	<0.001
Dairy milk	Soymilk	6995 (43.1)	–0.12 ± 0.00	–3.5 ± 0.1	<0.001	–43 ± 1	–2.2 ± 0.1	<0.001
Almonds	Peanuts	315 (2.4)	0.00 ± 0.00	–0.1 ± 0.0	<0.001	–34 ± 4	–0.7 ± 0.1	<0.001
Shrimp	Cod	480 (2.6)	–0.07 ± 0.01	–0.9 ± 0.1	<0.001	–3 ± 0	–0.2 ± 0.0	<0.001
Beef^4^	Chicken/pork	3320 (19.8)	–0.74 ± 0.03	–9.6 ± 0.3	<0.001	–192 ± 7	–5.9 ± 0.2	<0.001

1 Single-item substitutions were made for individuals who consumed one of the foods in the first column above. If the individual consumed the food more than once on the 24-h recall day, only 1 instance, the one with the highest carbon footprint, was substituted. Changes in environmental impacts are averaged across the entire sample and include those individuals who did not consume these foods and therefore had zero change in impact. See Supplementary Table 9 for these foods and their substitutions. kg CO_2_-eq, kilograms of carbon dioxide equivalents; liter-eq, liter-equivalent.

2 Number and percentage of respondents who consumed this food.

3 *P* value determined from a paired *t* test.

4 This is the main substitution from Table 3 that is displayed again here for convenience.

## Discussion

We approached the topic of dietary shifts to improve sustainability with a simple strategy: identify the highest-impact foods, find substitutes that were culinarily and calorically equivalent, and assess the difference in environmental impacts if individuals changed just 1 item in their diet. We identified beef as the overall most impactful food, and ∼20% of individuals ate this on the recall day. In these individuals, substitutions would result in decreases of 48% and 30% in their dietary carbon and water scarcity footprints, respectively, and result in a small improvement in their overall diet quality. We also examined a single-item change for a number of other foods. Other than the impact on the water scarcity footprint by substituting out asparagus or almonds, none of these substitutions had as much impact as replacing beef. When averaged across the entire sample, including both consumers and nonconsumers of a given food, changes by individuals who consumed beef made the greatest impact on the overall dietary carbon and water scarcity footprints.

Although our study is different from others in the specific type of diet modification, its results are broadly consistent with previous work. For example, Hallström and colleagues (21) reviewed the literature on diet change for sustainability, which included mostly studies from Europe, and found that changing current diets could reduce GHGE by up to 50%. This would place our carbon footprint result within the range they found, although at the high end. Aleksandrowicz et al. (20) reviewed studies that investigated the environmental impacts of changing diets to a more sustainable dietary pattern. These studies were predominantly from Europe with a handful from the United States, Canada, and Australia. Changes from a baseline diet to a vegan or vegetarian diet resulted in median declines of GHGE by 45% and 31%, respectively. They also identified studies that simulated replacement of ruminant meat (e.g., beef or lamb) with monogastric meat (e.g., chicken or pork) and found a median GHGE decline of 21%. This is much lower than our results, but our finding of a 48% decline applied to just those individuals whose baseline diet included beef. When averaged across the entire population, we saw a 10% drop in GHGE, which is closer in concept to the average national diet studies reviewed previously.

Not many studies have assessed the water scarcity of whole diets (32–38). Comparison of our results with these studies is difficult because other authors did not use the same spatially explicit water scarcity assessment method, used different food group aggregations, or did not assess specific commodity substitutions, as we did (32–37). That said, both Hess et al. (34) and Ridoutt et al. (37) observed notable differences in the water scarcity footprint of specific foods within a group, as we found here and as we have shown previously (31).

A number of authors have pointed out the difficulty in changing diets, in particular reducing meat intake (39, 40). Here we studied the potential impact of a simple substitution, one that does not reduce meat consumption, per se, but rather just beef consumption. Granted, there will be many consumers who would still resist such a dietary shift. But by keeping it simple, this approach will be easy for individuals who are motivated to change their diet for environmental reasons. Not only is self-efficacy a key prerequisite for planned behavioral change (17, 18), but it is also an important factor for population-wide changes toward sustainable diets (41).

In addition to modeling realistically achievable dietary changes, a strength of our work comes from our methods for calculating environmental impacts, which included a comprehensive review of the food life cycle assessment literature (15) and a state-of-the-art estimation of water scarcity footprints (31).

These changes were, of course, hypothetical; we did not measure actual dietary changes in individuals. This limitation may be outweighed, though, by our ability to assess such changes with a nationally representative sample using dietary data from NHANES. Another possible limitation is that we used information from a single 24-h recall, so we have not described the usual diets of individuals. NHANES did collect information on a second diet recall day on most respondents. However, the frequency of consumption of beef did not appear to differ between the 2 diet recall days recorded in NHANES, both being around one-fifth of the sample (19.8% and 20.1% on days 1 and 2, respectively). Moreover, our overriding concern in this study was to consider the diets themselves and the impacts of a potential change on the environmental impacts associated with those diets. As a check to see that diet quality was not compromised with these changes, we chose substitutions that were closely related nutritionally and, for the main substitution of interest, examined the resulting change with an overall indicator of diet quality, the HEI, that was designed for the US population. Other indicators, such as the Alternative Healthy Eating Index, are not as widely accepted in the United States. However, recent research has shown that the relation between irrigated water use and diet quality may vary according to which of these indicators is chosen (42).

Another limitation is that we did not know the production source of the foods consumed by respondents, as NHANES does not provide this level of detail, nor is the geographic origin readily known by, or available to, consumers. Furthermore, our environmental impacts database for GHGE was based on available literature, which skews more toward European studies, so it may not be completely representative of the US food supply. In addition to geography, production technique and the exact species influence GHGE values. For example, the GHGE associated with wild-caught tiger prawns from Australia is >7 times that of wild-caught banana prawns from the same area (43), and southern pink shrimp from Senegal that is produced industrially is many times more impactful than that from artisanal production (44). Still, our database had 95 entries for beef, which makes us much more confident of these results than for items such as shrimp, in which the 14 observations for crustaceans included lobster, shrimp, and prawns with diverse production techniques and locales.

We saw “culinary equivalence” as a key criterion for our substitutions to ensure consumer acceptability. For beef, our strategy was based on substituting other animal foods—chicken, turkey, or pork—because we assumed that meat eaters would more likely substitute another flesh food for their beef. We considered studying plant substitutions for beef, but culinarily equivalent replacements were not commonplace during the years of our study. However, future studies can incorporate the new plant-based “meat analogues,” such as the plant-based burgers designed by Impossible Foods and Beyond Meat that have found substantial consumer acceptance (45). Recent research shows that the carbon footprint of the Beyond Burger (0.24 kg CO_2_-eq per 100 g) is much lower than that of ground beef (3.28 kg CO_2_-eq per 100 g) and somewhat lower than that of a turkey burger (0.26 kg CO_2_-eq per 100 g) (15, 46). For other foods, such as asparagus or almonds, our “culinarily equivalent” criterion was less likely to be met. Peas and asparagus are both typically dinnertime vegetables, but the similarity breaks down after that. In addition, many individuals have peanut allergies, so obviously they cannot substitute them for almonds.

We estimated the potential impact on GHGE that people can make with simple substitutions in their daily diets. At both population and individual levels, replacing beef clearly had the greatest environmental impact. At an individual level, replacing shrimp, almonds, and asparagus also had sizable environmental impacts. Although individual substitutions were the focus of our study, we are not implying that the onus for addressing the climate problem is exclusively, or even principally, on individuals. The changes needed to address our climate problems are major. They are needed across all sectors and along all levels of human organization from international agencies to federal and state governments to communities and households. Many individuals feel strongly about this and wish to change our climate problem through direct actions that they can control. This, in turn, can change social norms about both the seriousness of the problem and the potential solutions that can address it (41). Our study provides evidence that even simple steps can assist in these efforts.

## Supplementary Material

nqab338_Supplemental_File

## Data Availability

NHANES data described in this manuscript are available at https://wwwn.cdc.gov/nchs/nhanes/. Data on the GHGE of foods are available at http://css.umich.edu/page/datafield. Data on the GHGE of diets are available at https://sph.tulane.edu/gchb/diet-environmental-impacts.

## Notes

Supported by the Wellcome Trust (grant 106854/Z/15/Z). Wellcome had no role whatsoever in the design, implementation, analysis, or interpretation of data. Funding for publication was provided, in part, by Tulane's Supporting Impactful Publications (SIP) Program.

Supplemental Tables 1–9 are available from the “Supplementary data” link in the online posting of the article and from the same link in the online table of contents at https://academic.oup.com/ajcn/.

Abbreviations used: AWARE, available water remaining; dataFIELD, database of Food Impacts on the Environment for Linking to Diets; FCID, Food Commodity Intake Database; FPED, Food Patterns Equivalents Database; GHGE, greenhouse gas emissions; HEI, Healthy Eating Index; kg CO_2_-eq, kilograms of carbon dioxide equivalents; liter-eq, liter-equivalent.
