# Supplementary material for: Single-item substitutions can substantially reduce the carbon and water scarcity footprints of US diets
Source: Am J Clin Nutr. 2022 Jan 13;115(2):378–87. doi: 10.1093/ajcn/nqab338 (PMC8827079; doi:10.1093/ajcn/nqab338)
Supplement: nqab338_Supplemental_File [file nqab338_supplemental_file.docx]

**On-line Supplementary Material**

**Single item substitutions can substantially reduce the carbon**

**and water scarcity footprints of US diets**

Donald Rose, Amelia M. Willits-Smith, Martin C. Heller

Supplementary Table 1. Point allocation in the 2010 Healthy Eating Index (HEI)

Supplementary Table 2. Highest ranked foods on overall diet carbon footprint

Supplementary Table 3. Highest ranked foods on consumed intensity of GHGE

Supplementary Table 4. Foods that were highest ranked on overall dietary GHGE impact and on consumed intensity of GHGE

Supplementary Table 5. Descriptions of original foods and their substitutions

Supplementary Table 6. Highest ranked foods on overall diet water scarcity footprint (WSF)

Supplementary Table 7. Highest ranked foods on consumed water scarcity intensity

Supplementary Table 8. Foods that ranked highest on overall water scarcity footprint and on consumed water scarcity intensity

Supplementary Table 9. Descriptions of additional foods and their substitutions

**Supplementary Table 1.** **Point allocation in the 2010 Healthy Eating Index (HEI)^1^**

|  | **Dietary component** | **Maximum Points^2^** | **Standard for maximum points^2^** | **Standard for minimum, or zero points ^2^** |
| --- | --- | --- | --- | --- |
| Adequacy**^3^** | Total Fruit | 5 | ≥ 0·8 c-eq/1000 kcal | No fruit |
|  | Whole Fruit | 5 | ≥ 0·4 c-eq/1000 kcal | No whole fruit |
|  | Total Vegetables | 5 | ≥ 1·1 c-eq/1000 kcal | No vegetables |
|  | Dark Greens and Legumes | 5 | ≥ 0·2 c-eq/1000 kcal | No dark-green vegies, beans, or peas |
|  | Whole Grains | 10 | ≥ 1·5 oz-eq/1000 kcal | No whole grains |
|  | Dairy | 10 | ≥ 1·3 c-eq/1000 kcal | No dairy |
|  | Total Protein Foods | 5 | ≥ 2·5 oz-eq/1000 kcal | No protein foods |
|  | Seafood and Plant Proteins | 5 | ≥ 0·8 c-eq/1000 kcal | No seafood or plant proteins |
|  | Fatty Acid Ratio**^4^** | 10 | (PUFAs+MUFAs)/SFAs ≥ 2·5 | (PUFAs+MUFAs)/SFAs ≤ 1·2 |
| Moderation**^5^** | Refined Grains | 10 | ≤ 1·8 oz-eq/1000 kcal | ≥ 4·3 oz-eq/1000 kcal |
|  | Sodium | 10 | ≤ 1·1 g/1000 kcal | ≥ 2·0 g/1000 kcal |
|  | Empty Calories | 20 | ≤ 19% of energy | ≥ 50% of energy |

^1^ Adapted from: Guenther PM, Casavale KO, Reedy J, et al. Update of the Healthy Eating Index: HEI-2010. *J Acad Nutr Diet* 2013; **113**(4): 569–80. Abbreviations: C-eq, cup equivalent; oz-eq = ounce equivalent; PUFAs, poly-unsaturated fatty acids; MUFAs, mono-unsaturated fatty acids; SFAs, saturated fatty acids.

**^2^** Points are allocated based on linear interpolation between specific densities (quantity/1000 kcal) or ratios that are assigned for minimum (zero) and maximum point totals.

**^3^** Higher score indicates higher consumption.

**^4^** Ratio of poly- and mono-unsaturated fatty acids to saturated fatty acids.

**^5^** Higher score indicates lower consumption.

| **Supplementary Table 2. Highest ranked foods on overall diet carbon footprint** | | |
| --- | --- | --- |
| **Code** | **Description** | **Diet carbon footprint**  **kgCO_2_-eq** |
| 21500100 | Ground beef or patty, cooked, NS as to percent lean | 54,342,094 |
| 21101130 | Beef steak, broiled or baked, lean only eaten | 37,218,816 |
| 21101120 | Beef steak, broiled or baked, lean and fat eaten | 19,721,155 |
| 11112110 | Milk, cow's, fluid, 2% fat | 17,952,170 |
| 25210210 | Frankfurter or hot dog, beef | 14,954,720 |
| 11111000 | Milk, cow's, fluid, whole | 11,949,858 |
| 21501300 | Ground beef, 85% - 89% lean, cooked (formerly extra lean) | 11,943,444 |
| 21501200 | Ground beef, 80% - 84% lean, cooked (formerly lean) | 10,484,747 |
| 58132310 | Spaghetti with tomato sauce and meatballs, meat sauce, or both | 9,965,872 |
| 27111410 | Chili con carne with beans | 9,457,485 |
| 11113000 | Milk, cow's, fluid, skim or nonfat, 0.5% or less butterfat | 9,455,669 |
| 92101000 | Coffee, made from ground, regular | 9,061,222 |
| 25231110 | Beef, sliced, prepackaged or deli, luncheon meat | 8,735,600 |
| 21401120 | Beef, roast, roasted, lean only eaten | 8,458,068 |
| 27214100 | Meat loaf made with beef | 8,373,804 |
| 27510330 | Double cheeseburger (2 patties), with tomato and/or catsup, on bun | 8,326,373 |
| 14104010 | Cheese, natural, Cheddar or American type | 8,169,308 |
| 25230210 | Ham, sliced, prepackaged or deli, luncheon meat | 7,730,904 |
| 21102130 | Beef steak, fried, lean only eaten | 7,413,691 |
| 14410200 | Cheese, processed, American or Cheddar type | 7,356,887 |
| 11112210 | Milk, cow's, fluid, 1% fat | 7,107,835 |
| 27116200 | Beef with barbecue sauce (mixture) | 6,751,980 |
| 13110100 | Ice cream, regular, flavors other than chocolate | 6,637,780 |
| 93101000 | Beer | 6,117,795 |
| 25221500 | Salami, NFS | 5,842,612 |
| 27214110 | Meat loaf made with beef, with tomato-based sauce | 5,568,381 |
| 26319140 | Shrimp, floured, breaded, or battered, fried | 5,436,636 |
| 93102000 | Beer, lite | 5,434,838 |
| 75652010 | Vegetable beef soup, home recipe | 5,322,505 |
| 21420100 | Beef, sandwich steak (flaked, formed, thinly sliced) | 5,276,770 |
| 21501000 | Ground beef, less than 80% lean, cooked (formerly regular) | 5,156,820 |
| 14010100 | Cheese, Cheddar or American type, NS as to natural or processed | 4,823,749 |
| 27212100 | Beef and noodles with tomato-based sauce (mixture) | 4,761,463 |
| 21102120 | Beef steak, fried, lean and fat eaten | 4,710,385 |
| 26319130 | Shrimp, steamed or boiled | 4,691,962 |
| 26319110 | Shrimp, cooked, NS as to cooking method | 4,691,805 |
| 14010000 | Cheese, NFS | 4,599,254 |
| 27510300 | Double cheeseburger (2 patties), with mayonnaise, on double-decker bun | 4,366,240 |
| 27510560 | Hamburger, 1/4 lb meat, with mayonnaise, and tomato and/or catsup, on bun | 4,246,910 |
| 58130011 | Lasagna with meat | 4,150,581 |
| 14109010 | Cheese, Swiss | 4,115,757 |
| 58101300 | Taco or tostada with beef, cheese and lettuce | 4,077,624 |
| 14204010 | Cheese, cottage, lowfat (1-2% fat) | 4,037,248 |
| 63149010 | Watermelon, raw | 3,759,992 |
| 25220410 | Bologna, NFS | 3,753,881 |
| 58101400 | Soft taco with beef, cheese, and lettuce | 3,572,751 |
| 24122120 | Chicken, breast, roasted, broiled, or baked, skin not eaten | 3,565,200 |
| 21000100 | Beef, NS as to cut, cooked, NS as to fat eaten | 3,561,801 |
| 25221310 | Polish sausage | 3,513,627 |
| 58100100 | Burrito with beef, no beans | 3,485,435 |

| **Supplementary Table 2 (cont’d). Highest ranked foods on overall diet carbon footprint** | | |
| --- | --- | --- |
| **Code** | **Description** | **Diet carbon footprint**  **kgCO_2_-eq** |
| 21401000 | Beef, roast, roasted, NS as to fat eaten | 3,437,522 |
| 21104110 | Beef steak, battered, fried, NS as to fat eaten | 3,309,924 |
| 25230310 | Chicken or turkey loaf, prepackaged or deli, luncheon meat | 3,293,058 |
| 21501350 | Ground beef, 90% - 94% lean, cooked | 3,189,091 |
| 61210220 | Orange juice, canned, bottled or in a carton | 3,066,117 |
| 27513010 | Roast beef sandwich | 2,958,914 |
| 21101000 | Beef steak, NS as to cooking method, NS as to fat eaten | 2,793,119 |
| 64104010 | Apple juice | 2,786,268 |
| 27510310 | Cheeseburger with tomato and/or catsup, on bun | 2,771,854 |
| 26305160 | Crab, hard shell, steamed | 2,754,239 |
| 81101000 | Butter, stick, salted | 2,669,266 |
| 25210280 | Frankfurter or hot dog, meat and poultry | 2,600,416 |
| 27212350 | Beef stroganoff with noodles | 2,565,232 |
| 27111420 | Chili con carne without beans | 2,550,777 |
| 24198700 | Chicken patty, fillet, or tenders, breaded, cooked | 2,479,566 |
| 28310330 | Beef and rice noodle soup, Oriental style (Vietnamese Pho Bo) | 2,464,792 |
| 58106530 | Pizza with meat, thick crust | 2,464,267 |
| 21407120 | Beef, pot roast, braised or boiled, lean only eaten | 2,445,849 |
| 21401110 | Beef, roast, roasted, lean and fat eaten | 2,411,181 |
| 21602100 | Beef jerky | 2,355,365 |
| 27111500 | Beef sloppy joe (no bun) | 2,319,818 |
| 14107010 | Cheese, Mozzarella, NFS | 2,287,157 |
| 27411200 | Beef with vegetables, tomato-based sauce | 2,256,351 |
| 58106720 | Pizza with meat and vegetables, thin crust | 2,249,226 |
| 25210220 | Frankfurter or hot dog, beef and pork | 2,239,441 |
| 58106520 | Pizza with meat, thin crust | 2,229,892 |
| 27510690 | Double hamburger (2 patties, 1/4 lb meat each), with mayo and tomatoes | 2,198,129 |
| 14107030 | Cheese, Mozzarella, part skim | 2,191,466 |
| 21417110 | Beef brisket, cooked, lean and fat eaten | 2,191,060 |
| 58100130 | Burrito with beef and cheese, no beans | 2,185,878 |
| 25221410 | Pork sausage, fresh, bulk, patty or link, cooked | 2,178,216 |
| 93401010 | Wine, table, red | 2,163,671 |
| 31105000 | Egg, whole, fried | 2,157,300 |
| 24100000 | Chicken, NS as to part and cooking method, NS as to skin eaten | 2,155,506 |
| 21105130 | Beef steak, braised, lean only eaten | 2,116,615 |
| 27212150 | Beef goulash with noodles | 2,115,021 |
| 27112010 | Salisbury steak with gravy (mixture) | 2,114,284 |
| 93401020 | Wine, table, white | 2,039,492 |
| 27510350 | Cheeseburger, 1/4 lb meat, with mayo, and tomato and/or catsup, on bun | 2,025,754 |
| 58106500 | Pizza with meat, prepared from frozen, thin crust | 2,011,817 |
| 14108400 | Cheese, Provolone | 2,005,082 |
| 61210250 | Orange juice, with calcium added, canned, bottled or in a carton | 2,000,469 |
| 26319120 | Shrimp, baked or broiled | 1,969,230 |
| 58106555 | Pizza with pepperoni, regular crust | 1,958,369 |
| 32104900 | Egg omelet or scrambled egg, NS as to fat added in cooking | 1,915,236 |
| 26305110 | Crab, cooked, NS as to cooking method | 1,889,800 |
| 63107010 | Banana, raw | 1,889,128 |
| 21103130 | Beef steak, breaded or floured, baked or fried, lean only eaten | 1,879,631 |
| 27560910 | Cold cut submarine sandwich, with cheese, lettuce, tomato, and spread | 1,856,325 |
| 21105120 | Beef steak, braised, lean and fat eaten | 1,846,364 |

| **Supplementary Table 3. Highest ranked foods on consumed intensity of GHGE ^1^** | | |  |
| --- | --- | --- | --- |
| **Code** | **Description** | **Intensity^1^**  **kg CO_2_-eq/100g** | |
| 21602000 | Beef, dried, chipped, uncooked | 5.425 | |
| 21602100 | Beef jerky | 5.404 | |
| 26319130 | Shrimp, steamed or boiled | 5.125 | |
| 26319110 | Shrimp, cooked, NS as to cooking method | 5.125 | |
| 26305160 | Crab, hard shell, steamed | 5.125 | |
| 26305110 | Crab, cooked, NS as to cooking method | 5.125 | |
| 26311110 | Lobster, cooked, NS as to cooking method | 5.125 | |
| 26311120 | Lobster, baked or broiled | 5.125 | |
| 26311160 | Lobster, steamed or boiled | 5.125 | |
| 26319120 | Shrimp, baked or broiled | 5.125 | |
| 26319170 | Shrimp, dried | 5.125 | |
| 26319180 | Shrimp, canned | 5.125 | |
| 26309160 | Crayfish, boiled or steamed | 5.084 | |
| 26319120 | Shrimp, baked or broiled | 5.030 | |
| 26319120 | Shrimp, baked or broiled | 4.986 | |
| 26311120 | Lobster, baked or broiled | 4.984 | |
| 26319120 | Shrimp, baked or broiled | 4.958 | |
| 26319120 | Shrimp, baked or broiled | 4.943 | |
| 26305120 | Crab, baked or broiled | 4.853 | |
| 26305120 | Crab, baked or broiled | 4.853 | |
| 23150200 | Goat, fried | 4.750 | |
| 23000100 | Lamb, NS as to cut, cooked | 4.746 | |
| 23101010 | Lamb chop, NS as to cut, cooked, lean and fat eaten | 4.746 | |
| 23101020 | Lamb chop, NS as to cut, cooked, lean only eaten | 4.746 | |
| 23104000 | Lamb, loin chop, cooked, NS as to fat eaten | 4.746 | |
| 23104010 | Lamb, loin chop, cooked, lean and fat eaten | 4.746 | |
| 23104020 | Lamb, loin chop, cooked, lean only eaten | 4.746 | |
| 23108020 | Lamb, shoulder, cooked, lean only eaten | 4.746 | |
| 23110050 | Lamb, ribs, cooked, lean and fat eaten | 4.746 | |
| 23120100 | Lamb, roast, cooked, NS as to fat eaten | 4.746 | |
| 23120110 | Lamb, roast, cooked, lean and fat eaten | 4.746 | |
| 23120120 | Lamb, roast, cooked, lean only eaten | 4.746 | |
| 23132000 | Lamb, ground or patty, cooked | 4.746 | |
| 23150100 | Goat, boiled | 4.746 | |
| 23150250 | Goat, baked | 4.746 | |
| 23150300 | Goat ribs, cooked | 4.746 | |
| 27250040 | Crab cake | 4.507 | |
| 25150000 | Brains, cooked | 4.408 | |
| 27150230 | Shrimp scampi | 4.292 | |
| 27150230 | Shrimp scampi | 4.281 | |
| 27250040 | Crab cake | 4.262 | |
| 27250040 | Crab cake | 4.262 | |
| 25170110 | Tripe, cooked | 4.216 | |
| 21101130 | Beef steak, broiled or baked, lean only eaten | 4.211 | |
| 21101120 | Beef steak, broiled or baked, lean and fat eaten | 4.211 | |
| 21501300 | Ground beef, 85% - 89% lean, cooked (formerly extra lean) | 4.211 | |
| 21501000 | Ground beef, less than 80% lean, cooked (formerly regular) | 4.211 | |
| 21000100 | Beef, NS as to cut, cooked, NS as to fat eaten | 4.211 | |
| 21401000 | Beef, roast, roasted, NS as to fat eaten | 4.211 | |

**^1^** Intensity estimates refer to the greenhouse gas emissions (GHGE) released in the production of food, adjusted for retail and consumer losses.

| **Supplementary Table 3 (cont’d). Highest ranked foods on consumed intensity of GHGE^1^** | | |
| --- | --- | --- |
| **Code** | **Description** | **Intensity^1^**  **kg CO_2_-eq/100g** |
| 21101000 | Beef steak, NS as to cooking method, NS as to fat eaten | 4.211 |
| 21407120 | Beef, pot roast, braised or boiled, lean only eaten | 4.211 |
| 21401110 | Beef, roast, roasted, lean and fat eaten | 4.211 |
| 21417110 | Beef brisket, cooked, lean and fat eaten | 4.211 |
| 21105130 | Beef steak, braised, lean only eaten | 4.211 |
| 21105120 | Beef steak, braised, lean and fat eaten | 4.211 |
| 21000110 | Beef, NS as to cut, cooked, lean and fat eaten | 4.211 |
| 21000120 | Beef, NS as to cut, cooked, lean only eaten | 4.211 |
| 21101010 | Beef steak, NS as to cooking method, lean and fat eaten | 4.211 |
| 21101020 | Beef steak, NS as to cooking method, lean only eaten | 4.211 |
| 21101110 | Beef steak, broiled or baked, NS as to fat eaten | 4.211 |
| 21105110 | Beef steak, braised, NS as to fat eaten | 4.211 |
| 21301000 | Beef, oxtails, cooked | 4.211 |
| 21302000 | Beef, neck bones, cooked | 4.211 |
| 21304000 | Beef, shortribs, cooked, NS as to fat eaten | 4.211 |
| 21304110 | Beef, shortribs, cooked, lean and fat eaten | 4.211 |
| 21304120 | Beef, shortribs, cooked, lean only eaten | 4.211 |
| 21305000 | Beef, cow head, cooked | 4.211 |
| 21401400 | Beef, roast, canned | 4.211 |
| 21407000 | Beef, pot roast, braised or boiled, NS as to fat eaten | 4.211 |
| 21407110 | Beef, pot roast, braised or boiled, lean and fat eaten | 4.211 |
| 21410000 | Beef, stew meat, cooked, NS as to fat eaten | 4.211 |
| 21410120 | Beef, stew meat, cooked, lean only eaten | 4.211 |
| 21417100 | Beef brisket, cooked, NS as to fat eaten | 4.211 |
| 21417120 | Beef brisket, cooked, lean only eaten | 4.211 |
| 23200100 | Veal, NS as to cut, cooked, NS as to fat eaten | 4.211 |
| 23210030 | Veal, roasted, lean only eaten | 4.211 |
| 23326100 | Bison, cooked | 4.211 |
| 21501200 | Ground beef, 80% - 84% lean, cooked (formerly lean) | 4.211 |
| 21401120 | Beef, roast, roasted, lean only eaten | 4.211 |
| 21501350 | Ground beef, 90% - 94% lean, cooked | 4.211 |
| 21501360 | Ground beef, 95% or more lean, cooked | 4.211 |
| 21420100 | Beef, sandwich steak (flaked, formed, thinly sliced) | 4.211 |
| 21500100 | Ground beef or patty, cooked, NS as to percent lean | 4.210 |
| 21500300 | Ground beef patty, cooked (for fast food sandwiches) | 4.210 |
| 23200120 | Veal, NS as to cut, cooked, lean only eaten | 4.190 |
| 23204030 | Veal cutlet or steak, NS as to cooking method, lean only eaten | 4.190 |
| 23204220 | Veal cutlet or steak, broiled, lean only eaten | 4.190 |
| 23204210 | Veal cutlet or steak, broiled, lean and fat eaten | 4.189 |
| 21416120 | Corned beef, cooked, lean only eaten | 4.170 |
| 21416150 | Corned beef, canned, ready-to-eat | 4.170 |
| 21601000 | Beef, bacon, cooked | 4.153 |
| 21416000 | Corned beef, cooked, NS as to fat eaten | 4.150 |
| 21416110 | Corned beef, cooked, lean and fat eaten | 4.150 |
| 23201030 | Veal chop, NS as to cooking method, lean only eaten | 4.125 |
| 21102120 | Beef steak, fried, lean and fat eaten | 4.113 |
| 21102110 | Beef steak, fried, NS as to fat eaten | 4.113 |
| 21003000 | Beef, NS as to cut, fried, NS to fat eaten | 4.103 |
| 21102130 | Beef steak, fried, lean only eaten | 4.098 |
| 25110140 | Beef liver, fried | 4.095 |
| 11830150 | Cocoa powder, not reconstituted (no dry milk) | 4.089 |

**^1^** Intensity estimates refer to the greenhouse gas emissions (GHGE) released in the production of food, adjusted for retail and consumer losses.

**Supplementary Table 4.** **Foods that were highest ranked on overall dietary GHGE impact and on consumed intensity of GHGE^1^**

| **Rank** | **Food Code** | **Food Description** | **Overall GHGE Impact (kg CO_2_-eq)** | **Intensity (kg CO_2_-eq/100g)** | **# people reporting** | **Amount Consumed (kg)** | **Overall Impact Rank** | **Intensity Rank** |
| --- | --- | --- | --- | --- | --- | --- | --- | --- |
| 1 | 21500100 | Ground beef or patty, cooked, NS as to percent lean | 54,342,094 | 4.210 | 987 | 1,290,648 | 1 | 83 |
| 2 | 21101130 | Beef steak, broiled or baked, lean only eaten | 37,218,816 | 4.211 | 477 | 883,883 | 2 | 44 |
| 3 | 21101120 | Beef steak, broiled or baked, lean and fat eaten | 19,721,155 | 4.211 | 166 | 468,344 | 3 | 45 |
| 4 | 21501300 | Ground beef, 85% - 89% lean, cooked | 11,943,444 | 4.211 | 146 | 283,636 | 7 | 46 |
| 5 | 21501200 | Ground beef, 80% - 84% lean, cooked (formerly lean) | 10,484,747 | 4.211 | 153 | 248,995 | 8 | 78 |
| 6 | 21401120 | Beef, roast, roasted, lean only eaten | 8,458,068 | 4.211 | 133 | 200,865 | 14 | 79 |
| 7 | 21102130 | Beef steak, fried, lean only eaten | 7,413,691 | 4.098 | 147 | 180,889 | 19 | 98 |
| 8 | 21420100 | Beef, sandwich steak (flaked, formed, thinly sliced) | 5,276,770 | 4.211 | 102 | 125,318 | 30 | 82 |
| 9 | 21501000 | Ground beef, less than 80% lean, cooked | 5,156,820 | 4.211 | 85 | 122,466 | 31 | 47 |
| 10 | 21102120 | Beef steak, fried, lean and fat eaten | 4,710,385 | 4.113 | 65 | 114,518 | 34 | 95 |
| 11 | 26319130 | Shrimp, steamed or boiled | 4,691,962 | 5.125 | 64 | 91,542 | 35 | 3 |
| 12 | 26319110 | Shrimp, cooked, NS as to cooking method | 4,691,805 | 5.125 | 112 | 91,539 | 36 | 4 |
| 13 | 21000100 | Beef, NS as to cut, cooked, NS as to fat eaten | 3,561,801 | 4.211 | 124 | 84,587 | 48 | 48 |
| 14 | 21401000 | Beef, roast, roasted, NS as to fat eaten | 3,437,522 | 4.211 | 68 | 81,635 | 51 | 49 |
| 15 | 21501350 | Ground beef, 90% - 94% lean, cooked | 3,189,091 | 4.211 | 41 | 75,735 | 54 | 80 |
| 16 | 21101000 | Beef steak, NS as to cooking method, NS as to fat eaten | 2,793,119 | 4.211 | 58 | 66,332 | 57 | 50 |
| 17 | 26305160 | Crab, hard shell, steamed | 2,754,239 | 5.125 | 37 | 53,736 | 60 | 5 |
| 18 | 21407120 | Beef, pot roast, braised or boiled, lean only eaten | 2,445,849 | 4.211 | 30 | 58,085 | 68 | 51 |
| 19 | 21401110 | Beef, roast, roasted, lean and fat eaten | 2,411,181 | 4.211 | 32 | 57,261 | 69 | 52 |
| 20 | 21602100 | Beef jerky | 2,355,365 | 5.404 | 78 | 43,582 | 70 | 2 |
| 21 | 21417110 | Beef brisket, cooked, lean and fat eaten | 2,191,060 | 4.211 | 12 | 52,034 | 79 | 53 |
| 22 | 21105130 | Beef steak, braised, lean only eaten | 2,116,615 | 4.211 | 46 | 50,266 | 85 | 54 |
| 23 | 26319120 | Shrimp, baked or broiled | 1,969,230 | 4.958 | 1 | 39,717 | 93 | 17 |
| 24 | 26305110 | Crab, cooked, NS as to cooking method | 1,889,800 | 5.125 | 24 | 36,871 | 96 | 6 |
| 25 | 21105120 | Beef steak, braised, lean and fat eaten | 1,846,364 | 4.211 | 17 | 43,848 | 100 | 55 |

**^1^** Foods in this list were in both top 100 lists shown in Supplementary Tables 2 and 3. They are ranked here by overall impact.

| **Supplementary Table 5. Descriptions of original foods and their substitutions** | | | |
| --- | --- | --- | --- |
| **Code** | **Description of Original Food** | **Code** | **Description of Substitution** |
| 21000100 | Beef, NS as to cut, cooked, NS as to fat eaten | 24100000 | Chicken, NS as to part and cooking method, NS as to skin eaten |
| 21000110 | Beef, NS as to cut, cooked, lean and fat eaten | 24100010 | Chicken, NS as to part and cooking method, skin eaten |
| 21000120 | Beef, NS as to cut, cooked, lean only eaten | 24100020 | Chicken, NS as to part and cooking method, skin not eaten |
| 21001000 | Steak, NS as to type of meat, cooked, NS as to fat eaten | 24100000 | Chicken, NS as to part and cooking method, NS as to skin eaten |
| 21001010 | Steak, NS as to type of meat, cooked, lean and fat eaten | 24100010 | Chicken, NS as to part and cooking method, skin eaten |
| 21001020 | Steak, NS as to type of meat, cooked, lean only eaten | 24100020 | Chicken, NS as to part and cooking method, skin not eaten |
| 21003000 | Beef, NS as to cut, fried, NS to fat eaten | 24104000 | Chicken, NS as to part, fried, no coating, NS as to skin eaten |
| 21101000 | Beef steak, NS as to cooking method, NS as to fat eaten | 24100000 | Chicken, NS as to part and cooking method, NS as to skin eaten |
| 21101010 | Beef steak, NS as to cooking method, lean and fat eaten | 24100010 | Chicken, NS as to part and cooking method, skin eaten |
| 21101020 | Beef steak, NS as to cooking method, lean only eaten | 24100020 | Chicken, NS as to part and cooking method, skin not eaten |
| 21101110 | Beef steak, broiled or baked, NS as to fat eaten | 24102000 | Chicken, NS as to part, roasted, broiled, or baked, NS as to skin eaten |
| 21101120 | Beef steak, broiled or baked, lean and fat eaten | 24102010 | Chicken, NS as to part, roasted, broiled, or baked, skin eaten |
| 21101130 | Beef steak, broiled or baked, lean only eaten | 24102020 | Chicken, NS as to part, roasted, broiled, or baked, skin not eaten |
| 21102110 | Beef steak, fried, NS as to fat eaten | 24104000 | Chicken, NS as to part, fried, no coating, NS as to skin eaten |
| 21102120 | Beef steak, fried, lean and fat eaten | 24104010 | Chicken, NS as to part, fried, no coating, skin eaten |
| 21102130 | Beef steak, fried, lean only eaten | 24104020 | Chicken, NS as to part, fried, no coating, skin not eaten |
| 21103110 | Beef steak, breaded or floured, baked or fried, NS as to fat eaten | 24107000 | Chicken, NS as to part, coated, baked or fried, prepared with skin, NS as to skin/coating eaten |
| 21103120 | Beef steak, breaded or floured, baked or fried, lean and fat eaten | 24107010 | Chicken, NS as to part, coated, baked or fried, prepared with skin, skin/coating eaten |
| 21103130 | Beef steak, breaded or floured, baked or fried, lean only eaten | 24107050 | Chicken, NS as to part, coated, baked or fried, prepared skinless, coating eaten |
| 21104110 | Beef steak, battered, fried, NS as to fat eaten | 24107000 | Chicken, NS as to part, coated, baked or fried, prepared with skin, NS as to skin/coating eaten |
| 21104120 | Beef steak, battered, fried, lean and fat eaten | 24107010 | Chicken, NS as to part, coated, baked or fried, prepared with skin, skin/coating eaten |
| 21104130 | Beef steak, battered, fried, lean only eaten | 24107050 | Chicken, NS as to part, coated, baked or fried, prepared skinless, coating eaten |
| 21105110 | Beef steak, braised, NS as to fat eaten | 24103000 | Chicken, NS as to part, stewed, NS as to skin eaten |
| 21105120 | Beef steak, braised, lean and fat eaten | 24103010 | Chicken, NS as to part, stewed, skin eaten |
| 21105130 | Beef steak, braised, lean only eaten | 24103020 | Chicken, NS as to part, stewed, skin not eaten |
| 21301000 | Beef, oxtails, cooked | 24100000 | Chicken, NS as to part and cooking method, NS as to skin eaten |
| 21302000 | Beef, neck bones, cooked | 24202600 | Turkey, neck, cooked |
| 21304000 | Beef, shortribs, cooked, NS as to fat eaten | 22701000 | Pork, spareribs, cooked, NS as to fat eaten |
| 21304110 | Beef, shortribs, cooked, lean and fat eaten | 22701010 | Pork, spareribs, cooked, lean and fat eaten |
| 21304120 | Beef, shortribs, cooked, lean only eaten | 22701020 | Pork, spareribs, cooked, lean only eaten |
| 21304200 | Beef, shortribs, barbecued, with sauce, NS as to fat eaten | 22701030 | Pork, spareribs, barbecued, with sauce, NS as to fat eaten |
| 21304210 | Beef, shortribs, barbecued, with sauce, lean and fat eaten | 22701040 | Pork, spareribs, barbecued, with sauce, lean and fat eaten |
| 21304220 | Beef, shortribs, barbecued, with sauce, lean only eaten | 22701050 | Pork, spareribs, barbecued, with sauce, lean only eaten |
| 21305000 | Beef, cow head, cooked | 22705010 | Pork ears, tail, head, snout, miscellaneous parts, cooked |
| 21401000 | Beef, roast, roasted, NS as to fat eaten | 24102000 | Chicken, NS as to part, roasted, broiled, or baked, NS as to skin eaten |
| 21401110 | Beef, roast, roasted, lean and fat eaten | 24102010 | Chicken, NS as to part, roasted, broiled, or baked, skin eaten |
| 21401120 | Beef, roast, roasted, lean only eaten | 24102020 | Chicken, NS as to part, roasted, broiled, or baked, skin not eaten |
| 21401400 | Beef, roast, canned | 24102000 | Chicken, NS as to part, roasted, broiled, or baked, NS as to skin eaten |
| 21407000 | Beef, pot roast, braised or boiled, NS as to fat eaten | 24103000 | Chicken, NS as to part, stewed, NS as to skin eaten |
| 21407110 | Beef, pot roast, braised or boiled, lean and fat eaten | 24103010 | Chicken, NS as to part, stewed, skin eaten |

| **Supplementary Table 5 (cont’d). Descriptions of original foods and their substitutions** | | | |
| --- | --- | --- | --- |
| **Code** | **Description of Original Food** | **Code** | **Description of Substitution** |
| 21407120 | Beef, pot roast, braised or boiled, lean only eaten | 24103020 | Chicken, NS as to part, stewed, skin not eaten |
| 21410000 | Beef, stew meat, cooked, NS as to fat eaten | 24100000 | Chicken, NS as to part and cooking method, NS as to skin eaten |
| 21410120 | Beef, stew meat, cooked, lean only eaten | 24103020 | Chicken, NS as to part, stewed, skin not eaten |
| 21416000 | Corned beef, cooked, NS as to fat eaten | 24100000 | Chicken, NS as to part and cooking method, NS as to skin eaten |
| 21416110 | Corned beef, cooked, lean and fat eaten | 24100010 | Chicken, NS as to part and cooking method, skin eaten |
| 21416120 | Corned beef, cooked, lean only eaten | 24100020 | Chicken, NS as to part and cooking method, skin not eaten |
| 21416150 | Corned beef, canned, ready-to-eat | 24100000 | Chicken, NS as to part and cooking method, NS as to skin eaten |
| 21417100 | Beef brisket, cooked, NS as to fat eaten | 24100000 | Chicken, NS as to part and cooking method, NS as to skin eaten |
| 21417110 | Beef brisket, cooked, lean and fat eaten | 24100010 | Chicken, NS as to part and cooking method, skin eaten |
| 21417120 | Beef brisket, cooked, lean only eaten | 24100020 | Chicken, NS as to part and cooking method, skin not eaten |
| 21420100 | Beef, sandwich steak (flaked, formed, thinly sliced) | 24100000 | Chicken, NS as to part and cooking method, NS as to skin eaten |
| 21500100 | Ground beef or patty, cooked, NS as to percent lean | 24207000 | Turkey, ground |
| 21500200 | Ground beef or patty, breaded, cooked | 24207000 | Turkey, ground |
| 21500300 | Ground beef patty, cooked (for fast food sandwiches) | 24207000 | Turkey, ground |
| 21501000 | Ground beef, less than 80% lean, cooked (formerly regular) | 24207000 | Turkey, ground |
| 21501200 | Ground beef, 80% - 84% lean, cooked (formerly lean) | 24207000 | Turkey, ground |
| 21501300 | Ground beef, 85% - 89% lean, cooked (formerly extra lean) | 24207000 | Turkey, ground |
| 21501350 | Ground beef, 90% - 94% lean, cooked | 24207000 | Turkey, ground |
| 21501360 | Ground beef, 95% or more lean, cooked | 24207000 | Turkey, ground |
| 21540100 | Ground beef with textured vegetable protein, cooked | 24207000 | Turkey, ground |
| 21601000 | Beef, bacon, cooked | 24208500 | Turkey bacon, cooked |
| 21602000 | Beef, dried, chipped, uncooked | 25230310 | Chicken or turkey loaf, prepackaged or deli, luncheon meat |
| 21602100 | Beef jerky | 22002800 | Pork jerky |
| 21603000 | Beef, pastrami (beef, smoked, spiced) | 25230820 | Turkey pastrami |

**Supplementary Table 6. Highest ranked foods on overall diet water scarcity footprint (WSF)^1^**

| **Code** | **Description** | **Overall WSF^1^**  **l-eq** |
| --- | --- | --- |
| 21500100 | Ground beef or patty, cooked, NS as to percent lean | 16,023,577,317 |
| 93401010 | Wine, table, red | 13,124,935,406 |
| 93401020 | Wine, table, white | 12,371,657,864 |
| 21101130 | Beef steak, broiled or baked, lean only eaten | 10,974,523,482 |
| 92101000 | Coffee, made from ground, regular | 10,238,761,466 |
| 61210220 | Orange juice, canned, bottled or in a carton | 8,454,707,196 |
| 93101000 | Beer | 6,078,095,932 |
| 63123000 | Grapes, raw, NS as to type | 5,834,913,721 |
| 21101120 | Beef steak, broiled or baked, lean and fat eaten | 5,815,076,374 |
| 61210250 | Orange juice, with calcium added, canned, bottled or in a carton | 5,516,220,201 |
| 93102000 | Beer, lite | 5,399,571,211 |
| 42104000 | Cashew nuts, NFS | 5,379,865,890 |
| 11112110 | Milk, cow's, fluid, 2% fat | 5,021,613,545 |
| 58132310 | Spaghetti with tomato sauce and meatballs, meat sauce or both | 4,883,182,558 |
| 63135010 | Peach, raw | 4,808,936,156 |
| 42116000 | Walnuts | 4,808,722,607 |
| 25210210 | Frankfurter or hot dog, beef | 4,419,859,528 |
| 64116020 | Grape juice | 4,338,511,267 |
| 42100100 | Almonds, NFS | 4,212,210,961 |
| 42110200 | Mixed nuts, dry roasted | 3,991,044,539 |
| 63109010 | Cantaloupe (muskmelon), raw | 3,967,952,686 |
| 42104200 | Cashew nuts, dry roasted | 3,877,987,618 |
| 92511010 | Fruit flavored drink (formerly lemonade) | 3,792,014,356 |
| 42110000 | Mixed nuts, NFS | 3,686,182,331 |
| 63105010 | Avocado, raw | 3,594,991,218 |
| 21501300 | Ground beef, 85% - 89% lean, cooked (formerly extra lean) | 3,521,702,658 |
| 11111000 | Milk, cow's, fluid, whole | 3,342,636,067 |
| 27111410 | Chili con carne with beans | 3,319,466,256 |
| 75202011 | Asparagus, cooked, from fresh, fat not added in cooking | 3,125,967,036 |
| 21501200 | Ground beef, 80% - 84% lean, cooked (formerly lean) | 3,091,584,309 |
| 63149010 | Watermelon, raw | 2,958,651,905 |
| 63101000 | Apple, raw | 2,934,643,626 |
| 42114130 | Pistachio nuts | 2,814,616,704 |
| 42501000 | Nut mixture with dried fruit and seeds | 2,794,480,706 |
| 11113000 | Milk, cow's, fluid, skim or nonfat, 0.5% or less butterfat | 2,644,956,638 |
| 25230210 | Ham, sliced, prepackaged or deli, luncheon meat | 2,618,106,369 |
| 27510330 | Double cheeseburger (2 patties), with tomato and/or catsup, on bun | 2,596,115,901 |
| 25231110 | Beef, sliced, prepackaged or deli, luncheon meat | 2,588,644,732 |
| 27214100 | Meat loaf made with beef | 2,518,334,784 |
| 21401120 | Beef, roast, roasted, lean only eaten | 2,493,987,685 |
| 63409010 | Guacamole, NFS | 2,421,444,937 |
| 72201211 | Broccoli, cooked, from fresh, fat not added in cooking | 2,250,561,990 |
| 61119010 | Orange, raw | 2,236,694,389 |
| 42101200 | Almonds, dry roasted (assume salted) | 2,235,451,799 |
| 21102130 | Beef steak, fried, lean only eaten | 2,189,432,953 |
| 27116200 | Beef with barbecue sauce (mixture) | 2,107,461,666 |
| 13110100 | Ice cream, regular, flavors other than chocolate | 2,082,975,688 |
| 64100110 | Fruit juice blend, 100% juice | 2,072,655,331 |
| 25230310 | Chicken or turkey loaf, prepackaged or deli, luncheon meat | 2,041,373,363 |
| 62125100 | Raisins | 2,023,972,866 |
| **^1^** Population level metric of irrigated water used to produce foods in the US diet, characterized by the relative scarcity of water at the source of production and adjusted for retail and consumer losses. | | |

| **Supplementary Table 6 (cont’d). Highest ranked foods on overall diet water scarcity footprint (WSF)** | | |
| --- | --- | --- |
| **Code** | **Description** | **Overall WSF**  **l-eq** |
| 11112210 | Milk, cow's, fluid, 1% fat | 1,988,216,487 |
| 75113000 | Lettuce, raw | 1,835,776,179 |
| 25221500 | Salami, NFS | 1,753,387,912 |
| 75652010 | Vegetable beef soup, home recipe | 1,751,372,281 |
| 64104010 | Apple juice | 1,737,813,593 |
| 63115010 | Cherries, sweet, raw (Queen Anne, Bing) | 1,717,749,832 |
| 27214110 | Meat loaf made with beef, with tomato-based sauce | 1,676,411,490 |
| 56205010 | Rice, white, cooked, regular, fat not added in cooking | 1,652,236,693 |
| 24122120 | Chicken, breast, roasted, broiled, or baked, skin not eaten | 1,601,318,229 |
| 74101000 | Tomatoes, raw | 1,588,643,165 |
| 63107010 | Banana, raw | 1,581,093,235 |
| 14104010 | Cheese, natural, Cheddar or American type | 1,577,170,075 |
| 21420100 | Beef, sandwich steak (flaked, formed, thinly sliced) | 1,555,934,395 |
| 63131010 | Nectarine, raw | 1,520,987,806 |
| 21501000 | Ground beef, less than 80% lean, cooked (formerly regular) | 1,520,565,283 |
| 63143010 | Plum, raw | 1,512,268,430 |
| 64100200 | Fruit juice blend, with cranberry, 100% juice | 1,488,171,861 |
| 58132110 | Spaghetti with tomato sauce, meatless | 1,446,757,663 |
| 27212100 | Beef and noodles with tomato-based sauce (mixture) | 1,446,080,884 |
| 92410310 | Soft drink, cola-type | 1,431,585,347 |
| 58130011 | Lasagna with meat | 1,397,470,221 |
| 21102120 | Beef steak, fried, lean and fat eaten | 1,390,797,184 |
| 91401000 | Jelly, all flavors | 1,386,875,637 |
| 57227000 | Granola, NFS | 1,384,917,740 |
| 27510300 | Double cheeseburger (2 patties), with mayonnaise or salad dressing, on double-decker bun | 1,360,550,159 |
| 63223020 | Strawberries, raw | 1,359,672,210 |
| 27510560 | Hamburger, 1/4 lb meat, with mayonnaise or salad dressing, and tomato and/or catsup, on bun | 1,330,094,454 |
| 61125010 | Tangerine, raw | 1,297,439,376 |
| 93404000 | Wine cooler | 1,294,993,114 |
| 75202021 | Asparagus, cooked, from fresh, w/ vegetable oil, NFS (Modification code: 205482) | 1,280,895,331 |
| 58101300 | Taco or tostada with beef, cheese and lettuce | 1,255,626,889 |
| 51150000 | Roll, white, soft | 1,252,974,976 |
| 24198700 | Chicken patty, fillet, or tenders, breaded, cooked | 1,235,469,093 |
| 64126000 | Pomegranate juice | 1,197,793,319 |
| 75510000 | Olives, NFS | 1,177,997,589 |
| 14410200 | Cheese, processed, American or Cheddar type | 1,167,719,388 |
| 58101400 | Soft taco with beef, cheese, and lettuce | 1,151,680,254 |
| 73101010 | Carrots, raw | 1,139,656,216 |
| 25220410 | Bologna, NFS | 1,131,589,991 |
| 63129010 | Mango, raw | 1,121,201,725 |
| 58106720 | Pizza with meat and vegetables, thin crust | 1,085,875,737 |
| 58100100 | Burrito with beef, no beans | 1,075,750,795 |
| 25221310 | Polish sausage | 1,056,652,771 |
| 21000100 | Beef, NS as to cut, cooked, NS as to fat eaten | 1,050,250,195 |
| 75117020 | Onions, mature, raw | 1,043,074,234 |
| 64132010 | Prune juice | 1,032,467,162 |
| 41205010 | Refried beans | 1,025,976,010 |
| 51101000 | Bread, white | 1,023,949,815 |
| 21401000 | Beef, roast, roasted, NS as to fat eaten | 1,013,604,453 |
| 58106530 | Pizza with meat, thick crust | 1,003,091,226 |
| **^1^** Population level metric of irrigated water used to produce foods in the US diet, characterized by the relative scarcity of water at the source of production and adjusted for retail and consumer losses. | | |

**Supplementary Table 7. Highest ranked foods on consumed water scarcity intensity^1^**

| **Code** | **Description** | **Intensity**  **l-eq/100g** |
| --- | --- | --- |
| 42116000 | Walnuts | 8,306 |
| 42114130 | Pistachio nuts | 7,986 |
| 42201000 | Cashew butter | 7,536 |
| 42104200 | Cashew nuts, dry roasted | 7,531 |
| 42104205 | Cashew nuts, dry roasted, without salt | 7,531 |
| 42104110 | Cashew nuts, roasted, without salt | 7,521 |
| 42104000 | Cashew nuts, NFS | 7,403 |
| 42104100 | Cashew nuts, roasted (assume salted) | 7,403 |
| 42116100 | Walnuts, honey-roasted | 6,573 |
| 42104500 | Cashew nuts, honey-roasted | 6,365 |
| 42100100 | Almonds, NFS | 5,074 |
| 42101000 | Almonds, unroasted | 5,074 |
| 42101210 | Almonds, dry roasted, without salt | 5,074 |
| 42200500 | Almond butter | 5,017 |
| 42101200 | Almonds, dry roasted (assume salted) | 4,974 |
| 42101100 | Almonds, roasted | 4,698 |
| 42110200 | Mixed nuts, dry roasted | 4,622 |
| 42110100 | Mixed nuts, roasted, with peanuts | 4,410 |
| 42100050 | Nuts, nfs | 4,410 |
| 42110000 | Mixed nuts, NFS | 4,410 |
| 42109100 | Macadamia nuts, roasted | 4,379 |
| 42101350 | Almonds, honey-roasted | 4,139 |
| 62104100 | Apricot, dried, uncooked | 4,000 |
| 92307000 | Tea, powdered instant, unsweetened, dry | 3,987 |
| 42110300 | Mixed nuts, honey-roasted, with peanuts | 3,838 |
| 42102000 | Brazil nuts | 3,811 |
| 91701010 | Almonds, chocolate covered | 3,681 |
| 42112000 | Pecans | 3,654 |
| 62113100 | Fig, dried, uncooked | 3,553 |
| 62110100 | Date | 3,464 |
| 62122100 | Prune, dried, uncooked | 3,431 |
| 42200600 | Almond paste (Marzipan paste) | 3,016 |
| 75100800 | Asparagus, raw | 2,917 |
| 75202010 | Asparagus, cooked, NS as to form, fat not added in cooking | 2,917 |
| 75202011 | Asparagus, cooked, from fresh, fat not added in cooking | 2,917 |
| 75202012 | Asparagus, cooked, from frozen, fat not added in cooking | 2,917 |
| 75202013 | Asparagus, cooked, from canned, fat not added in cooking | 2,917 |
| 75202023 | Asparagus, cooked, from canned, w/ butter, NFS (Modification code: 100156) | 2,868 |
| 75202023 | Asparagus, cooked, from canned, fat added in cooking | 2,864 |
| 75202021 | Asparagus, cooked, from fresh, cooking w/ butter, NFS (Modification code: 200394) | 2,851 |
| 75202000 | Asparagus, cooked, NS as to form, NS as to fat added in cooking | 2,847 |
| 75202001 | Asparagus, cooked, from fresh, NS as to fat added in cooking | 2,847 |
| 75202020 | Asparagus, cooked, NS as to form, fat added in cooking | 2,847 |
| 75202021 | Asparagus, cooked, from fresh, fat added in cooking | 2,847 |
| 91304060 | Topping, nut (wet) | 2,845 |
| 75202021 | Asparagus, cooked, from fresh, w/ vegetable oil, NFS (Modification code: 205482) | 2,843 |
| 62116100 | Peach, dried, uncooked | 2,523 |
| 91728500 | Sugared pecans (sugar and egg white coating) | 2,517 |
| 75201011 | Artichoke, globe (French), cooked, from fresh, fat not added in cooking | 2,371 |
| 91701030 | Almonds, yogurt-covered | 2,362 |
| **^1^** Irrigated water used to produce a food, characterized by the relative scarcity of water at the source of production and adjusted for retail and consumer losses. | |  |

1

| **Supplementary Table 7 (cont’d). Highest ranked foods on consumed water scarcity intensity^1^** | | |
| --- | --- | --- |
| **Code** | **Description** | **Intensity**  **l-eq/100g^1^** |
| 42501000 | Nut mixture with dried fruit and seeds | 2,355 |
| 75201000 | Artichoke, globe (French), cooked, NS as to form, NS as to fat added in cooking | 2,311 |
| 75201021 | Artichoke, globe (French), cooked, from fresh, fat added in cooking | 2,311 |
| 91727010 | Nuts, chocolate covered, not almonds or peanuts | 2,309 |
| 75201021 | Artichoke, globe (French), cooked, from fresh, w/ vegetable oil, NFS (Modification code: 206492) | 2,309 |
| 62116230 | Peach, dried, cooked, with sugar | 2,302 |
| 75201013 | Artichoke, globe (French), cooked, from canned, fat not added in cooking | 2,298 |
| 75201030 | Artichoke salad in oil | 2,186 |
| 62125100 | Raisins | 2,098 |
| 75401011 | Asparagus, from fresh, creamed or with cheese sauce | 1,982 |
| 75401012 | Asparagus, from frozen, creamed or with cheese sauce | 1,982 |
| 91406600 | Jams, preserves, marmalades, low sugar (all flavors) | 1,981 |
| 42113000 | Pine nuts (Pignolias) | 1,906 |
| 62101000 | Fruit, dried, NFS (assume uncooked) | 1,898 |
| 62101050 | Fruit mixture, dried (includes 3+ of: apple, apricot, date, papaya, peach, pear, pineapple, prune, raisin) | 1,898 |
| 91700500 | M&M's Almond Chocolate Candies | 1,875 |
| 91701020 | Almonds, sugar-coated | 1,756 |
| 53248000 | Cookie, whole wheat, dried fruit, nut | 1,679 |
| 21602000 | Beef, dried, chipped, uncooked | 1,600 |
| 21602100 | Beef jerky | 1,595 |
| 42107000 | Filberts, hazelnuts | 1,543 |
| 62114050 | Mango, dried | 1,450 |
| 64126000 | Pomegranate juice | 1,391 |
| 53441110 | Baklava | 1,388 |
| 91708070 | Tamarind candy | 1,353 |
| 91703500 | Nuts, carob-coated | 1,347 |
| 61204000 | Lemon juice, NS as to form | 1,327 |
| 61204010 | Lemon juice, freshly squeezed | 1,327 |
| 61204200 | Lemon juice, canned or bottled | 1,327 |
| 61204600 | Lemon juice, frozen | 1,327 |
| 61207000 | Lime juice, NS as to form | 1,327 |
| 61207010 | Lime juice, freshly squeezed | 1,327 |
| 23150200 | Goat, fried | 1,326 |
| 23000100 | Lamb, NS as to cut, cooked | 1,323 |
| 23101010 | Lamb chop, NS as to cut, cooked, lean and fat eaten | 1,323 |
| 23101020 | Lamb chop, NS as to cut, cooked, lean only eaten | 1,323 |
| 23104000 | Lamb, loin chop, cooked, NS as to fat eaten | 1,323 |
| 23104010 | Lamb, loin chop, cooked, lean and fat eaten | 1,323 |
| 23104020 | Lamb, loin chop, cooked, lean only eaten | 1,323 |
| 23108020 | Lamb, shoulder, cooked, lean only eaten | 1,323 |
| 23110050 | Lamb, ribs, cooked, lean and fat eaten | 1,323 |
| 23120100 | Lamb, roast, cooked, NS as to fat eaten | 1,323 |
| 23120110 | Lamb, roast, cooked, lean and fat eaten | 1,323 |
| 23120120 | Lamb, roast, cooked, lean only eaten | 1,323 |
| 23132000 | Lamb, ground or patty, cooked | 1,323 |
| 23150100 | Goat, boiled | 1,323 |
| 23150250 | Goat, baked | 1,323 |
| 23150300 | Goat ribs, cooked | 1,323 |
| 75510000 | Olives, NFS | 1,287 |
| 75510010 | Olives, green | 1,287 |
| 75510020 | Olives, black | 1,287 |

**^1^** See footnote on page S-12.

**Supplementary Table 8.** **Foods that ranked highest on overall water scarcity footprint and on consumed water scarcity intensity^1^**

| **Rank** | **Food code** | **Description** | **Overall WSF impact (l-eq)** | **Consumed water scarcity intensity (l-eq /100g)^1^** | **Overall impact rank** | **Intensity rank** | **# people reporting** | **# times reported** |
| --- | --- | --- | --- | --- | --- | --- | --- | --- |
| 1 | 42104000 | Cashew nuts, NFS | 5,379,865,890 | 7,403 | 12 | 7 | 78 | 78 |
| 2 | 42116000 | Walnuts | 4,808,722,607 | 8,306 | 16 | 1 | 156 | 163 |
| 3 | 42100100 | Almonds, NFS | 4,212,210,961 | 5,074 | 19 | 11 | 192 | 208 |
| 4 | 42110200 | Mixed nuts, dry roasted | 3,991,044,539 | 4,622 | 20 | 17 | 78 | 86 |
| 5 | 42104200 | Cashew nuts, dry roasted | 3,877,987,618 | 7,531 | 22 | 4 | 57 | 63 |
| 6 | 42110000 | Mixed nuts, NFS | 3,686,182,331 | 4,410 | 24 | 20 | 131 | 142 |
| 7 | 75202011 | Asparagus, cooked, from fresh, fat not added in cooking | 3,125,967,036 | 2,917 | 29 | 35 | 57 | 59 |
| 8 | 42114130 | Pistachio nuts | 2,814,616,704 | 7,986 | 33 | 2 | 60 | 62 |
| 9 | 42501000 | Nut mixture with dried fruit and seeds | 2,794,480,706 | 2,355 | 34 | 51 | 103 | 112 |
| 10 | 42101200 | Almonds, dry roasted (assume salted) | 2,235,451,799 | 4,974 | 44 | 15 | 58 | 62 |
| 11 | 62125100 | Raisins | 2,023,972,866 | 2,098 | 50 | 59 | 239 | 249 |
| 12 | 75202021 | Asparagus, cooked, from fresh, fat added in cooking | 1,280,895,331 | 2,843 | 80 | 46 | 25 | 25 |
| 13 | 64126000 | Pomegranate juice | 1,197,793,319 | 1,391 | 84 | 73 | 20 | 21 |
| 14 | 75510000 | Olives, NFS | 1,177,997,589 | 1,287 | 85 | 99 | 249 | 254 |

**^1^** Foods in this list were in both top 100 lists, that for the overall population wide dietary water scarcity footprint and that for the water scarcity intensity (liter-equivalents per 100 grams of each food). They are ranked here by overall impact on water scarcity footprint. Consumed water intensity refers to the irrigated water used to produce a food, characterized by the relative scarcity of water at the source of production and adjusted for retail and consumer losses, and is expressed in liter-equivalents per 100 g.

**Supplementary Table 9. Descriptions of additional foods and their substitutions**

| **Code** | **Description of Additional Food** | **Code** | **Description of Substitution** | |
| --- | --- | --- | --- | --- |
| ***Soy milk for fluid milk*** | | | | |
| 11100000 | Milk, NFS | 11320000 | Milk, soy, ready-to-drink, not baby's | |
| 11111000 | Milk, cow's, fluid, whole | 11320000 | Milk, soy, ready-to-drink, not baby's | |
| 11111160 | Milk, calcium fortified, cow's, fluid, 1% fat | 11320000 | Milk, soy, ready-to-drink, not baby's | |
| 11111170 | Milk, calcium fortified, cow's, fluid, skim or nonfat | 11320100 | Milk, soy, light, ready-to-drink, not baby's | |
| 11112110 | Milk, cow's, fluid, 2% fat | 11320000 | Milk, soy, ready-to-drink, not baby's | |
| 11112120 | Milk, cow's, fluid, acidophilus, 1% fat | 11320000 | Milk, soy, ready-to-drink, not baby's | |
| 11112130 | Milk, cow's, fluid, acidophilus, 2% fat | 11320100 | Milk, soy, light, ready-to-drink, not baby's | |
| 11112210 | Milk, cow's, fluid, 1% fat | 11320200 | Milk, soy, nonfat, ready-to-drink, not baby's | |
| 11113000 | Milk, cow's, fluid, skim or nonfat, 0.5% or less butterfat | 11320100 | Milk, soy, light, ready-to-drink, not baby's | |
| 11114300 | Milk, cow's, fluid, lactose reduced, 1% fat | 11320100 | Milk, soy, light, ready-to-drink, not baby's | |
| 11114320 | Milk, cow's, fluid, lactose reduced, nonfat | 11320000 | Milk, soy, ready-to-drink, not baby's | |
| 11114330 | Milk, cow's, fluid, lactose reduced, 2% fat | 11320100 | Milk, soy, light, ready-to-drink, not baby's | |
| 11114350 | Milk, cow's, fluid, lactose reduced, whole | 11320200 | Milk, soy, nonfat, ready-to-drink, not baby's | |
| 11120000 | Milk, dry, reconstituted, NFS | 11320200 | Milk, soy, nonfat, ready-to-drink, not baby's | |
| 11121100 | Milk, dry, reconstituted, whole | 11320000 | Milk, soy, ready-to-drink, not baby's | |
| 11121210 | Milk, dry, reconstituted, lowfat | 11320000 | Milk, soy, ready-to-drink, not baby's | |
| 11121300 | Milk, dry, reconstituted, nonfat | 11320200 | Milk, soy, nonfat, ready-to-drink, not baby's | |
| ***Peanuts for Almonds*** | | | | |
| 42100100 | Almonds, NFS | 42111000 | Peanuts, NFS | |
| 42101000 | Almonds, unroasted | 42111000 | Peanuts, NFS | |
| 42101100 | Almonds, roasted | 42111100 | Peanuts, roasted, salted | |
| 42101200 | Almonds, dry roasted (assume salted) | 42111200 | Peanuts, dry roasted, salted | |
| 42101210 | Almonds, dry roasted, without salt | 42111210 | Peanuts, dry roasted, without salt | |
| 42101350 | Almonds, honey-roasted | 42111500 | Peanuts, honey-roasted | |
| 42200500 | Almond butter | 42202000 | Peanut butter | |
| ***Whole Wheat Bread for White Bread*** | | | | |
| 51000100 | Bread, NS as to major flour | 51300110 | Bread, whole wheat, NS as to 100% | |
| 51000110 | Bread, NS as to major flour, toasted | 51208010 | Bagel, whole wheat, 100%, toasted |  |
| 51000180 | Bread, made from home recipe or purchased at a bakery, NS as to major flour | 51201060 | Bread, whole wheat, 100%, made from home recipe or purchased at bakery |  |
| 51000200 | Roll, NS as to major flour | 51220000 | Roll, whole wheat, 100% |  |
| 51000250 | Roll, made from home recipe or purchased at a bakery, NS as to major flour | 51220000 | Roll, whole wheat, 100% |  |
| 51101000 | Bread, white | 51201010 | Bread, whole wheat, 100% | |
| 51101010 | Bread, white, toasted | 51201020 | Bread, whole wheat, 100%, toasted | |

| **Supplementary Table 9 (cont’d). Descriptions of additional foods and their substitutions** | | | |
| --- | --- | --- | --- |
| **Code** | **Description of Additional Food** | **Code** | **Description of Substitution** |
| ***Whole Wheat Bread for White Bread (cont’d)*** | | | |
| 51101050 | Bread, white, made from home recipe or purchased at a bakery | 51201060 | Bread, whole wheat, 100%, made from home recipe or purchased at bakery |
| 51101060 | Bread, white, made from home recipe or purchased at a bakery, toasted | 51201060 | Bread, whole wheat, 100%, made from home recipe or purchased at bakery |
| 51105010 | Bread, Cuban | 51201010 | Bread, whole wheat, 100% |
| 51105040 | Bread, Cuban, toasted | 51201020 | Bread, whole wheat, 100%, toasted |
| 51107010 | Bread, French or Vienna | 51201010 | Bread, whole wheat, 100% |
| 51107040 | Bread, French or Vienna, toasted | 51201020 | Bread, whole wheat, 100%, toasted |
| 51108010 | Focaccia, Italian flatbread, plain | 51201010 | Bread, whole wheat, 100% |
| 51108100 | Naan, Indian flatbread | 51201010 | Bread, whole wheat, 100% |
| 51109010 | Bread, Italian, Grecian, Armenian | 51201010 | Bread, whole wheat, 100% |
| 51109040 | Bread, Italian, Grecian, Armenian, toasted | 51201020 | Bread, whole wheat, 100%, toasted |
| 51122000 | Bread, reduced calorie and/or high fiber, white or NFS | 51201010 | Bread, whole wheat, 100% |
| 51122010 | Bread, reduced calorie and/or high fiber, white or NFS, toasted | 51201020 | Bread, whole wheat, 100%, toasted |
| 51123020 | Bread, high protein, toasted | 51201020 | Bread, whole wheat, 100%, toasted |
| 51133010 | Bread, sour dough | 51201010 | Bread, whole wheat, 100% |
| 51133020 | Bread, sour dough, toasted | 51201020 | Bread, whole wheat, 100%, toasted |
| 51150000 | Roll, white, soft | 51220000 | Roll, whole wheat, 100% |
| 51150100 | Roll, white, soft, toasted | 51220000 | Roll, whole wheat, 100% |
| 51152000 | Roll, white, soft, reduced calorie and/or high fiber | 51220000 | Roll, whole wheat, 100% |
| 51153000 | Roll, white, hard | 51220000 | Roll, whole wheat, 100% |
| 51153010 | Roll, white, hard, toasted | 51220000 | Roll, whole wheat, 100% |
| 51155000 | Roll, French or Vienna | 51220000 | Roll, whole wheat, 100% |
| 51157000 | Roll, hoagie, submarine | 51220000 | Roll, whole wheat, 100% |
| 51158100 | Roll, Mexican, bolillo | 51220000 | Roll, whole wheat, 100% |
| 51159000 | Roll, sour dough | 51201010 | Bread, whole wheat, 100% |
| 51180010 | Bagel | 51208000 | Bagel, whole wheat, 100% |
| 51180020 | Bagel, toasted | 51208010 | Bagel, whole wheat, 100%, toasted |
| 51186010 | Muffin, English | 51303030 | Muffin, English, whole wheat, NS as to 100% |
| ***Bulgur Wheat for Rice*** | | | |
| 56204980 | Rice, white, cooked, converted, NS as to fat added in cooking | 56207130 | Bulgur, cooked or canned, NS as to fat added in cooking |
| 56204990 | Rice, white, cooked, regular, NS as to fat added in cooking | 56207130 | Bulgur, cooked or canned, NS as to fat added in cooking |
| 56205000 | Rice, cooked, NFS | 56207130 | Bulgur, cooked or canned, NS as to fat added in cooking |
| 56205010 | Rice, white, cooked, regular, fat not added in cooking | 56207110 | Bulgur, cooked or canned, fat not added in cooking |
| ***Bulgur Wheat for Rice (cont’d)*** | | | |
| 56205020 | Rice, white, cooked, instant, NS as to fat added in cooking | 56207130 | Bulgur, cooked or canned, NS as to fat added in cooking |
| 56205030 | Rice, white, cooked, instant, fat not added in cooking | 56207110 | Bulgur, cooked or canned, fat not added in cooking |
| 56205040 | Rice, white, cooked, converted, fat not added in cooking | 56207110 | Bulgur, cooked or canned, fat not added in cooking |
| 56205130 | Yellow rice, cooked, regular, NS as to fat added in cooking | 56207130 | Bulgur, cooked or canned, NS as to fat added in cooking |
| 56205150 | Yellow rice, cooked, regular, fat not added in cooking | 56207110 | Bulgur, cooked or canned, fat not added in cooking |
| 56205170 | Yellow rice, cooked, regular, fat added in cooking w/ butter, NFS (201429)^1^ | 56207120 | Bulgur, cooked or canned, fat added in cooking |
| 56205170 | Yellow rice, cooked, regular, fat added in cooking | 56207120 | Bulgur, cooked or canned, fat added in cooking |
| 56205170 | Yellow rice, cooked, regular, fat added in cooking w/ vegetable oil, NFS (200798) | 56207120 | Bulgur, cooked or canned, fat added in cooking w/ vegetable oil, NFS (204688) |
| 56205190 | Rice, white, cooked, glutinous | 56207130 | Bulgur, cooked or canned, NS as to fat added in cooking |
| 56205400 | Rice, cooked, NS as to type, fat added in cooking | 56207120 | Bulgur, cooked or canned, fat added in cooking |
| 56205400 | Rice, cooked, NS as to type, fat added in cooking w/ vegetable oil, NFS (200334) | 56207120 | Bulgur, cooked or canned, fat added in cooking w/ vegetable oil, NFS (204688) |
| 56205410 | Rice, white, cooked with (fat) oil, Puerto Rican style (Arroz blanco) | 56207120 | Bulgur, cooked or canned, fat added in cooking w/ vegetable oil, NFS (204688) |
| 56205420 | Rice, white, cooked, regular, w/ animal fat or meat drippings (101262) | 56207120 | Bulgur, cooked or canned, fat added in cooking |
| 56205420 | Rice, white, cooked, regular, fat added in cooking w/ butter, NFS (200056) | 56207120 | Bulgur, cooked or canned, fat added in cooking |
| 56205420 | Rice, white, cooked, regular, fat added in cooking | 56207120 | Bulgur, cooked or canned, fat added in cooking |
| 56205420 | Rice, white, cooked, regular, fat added in cooking w/ vegetable oil, NFS (200333) | 56207120 | Bulgur, cooked or canned, fat added in cooking w/ vegetable oil, NFS (204688) |
| 56205420 | Rice, white, cooked, regular, w/ vegetable shortening (201246) | 56207120 | Bulgur, cooked or canned, fat added in cooking |
| 56205430 | Rice, white, cooked, instant, fat added in cooking w/ butter, NFS (100022) | 56207120 | Bulgur, cooked or canned, fat added in cooking |
| 56205430 | Rice, white, cooked, instant, fat added in cooking | 56207120 | Bulgur, cooked or canned, fat added in cooking |
| 56205430 | Rice, white, cooked, instant, fat added in cooking w/ vegetable oil, NFS (205179) | 56207120 | Bulgur, cooked or canned, fat added in cooking w/ vegetable oil, NFS (204688) |
| 56205440 | Rice, white, cooked, converted, fat added in cooking w/ butter, NFS (200204) | 56207120 | Bulgur, cooked or canned, fat added in cooking |
| 56205440 | Rice, white, cooked, converted, fat added in cooking | 56207120 | Bulgur, cooked or canned, fat added in cooking |
| 56205440 | Rice, white, cooked, converted, w/ vegetable oil, NFS (100900) | 56207120 | Bulgur, cooked or canned, fat added in cooking w/ vegetable oil, NFS (204688) |
| ***Apples for Grapes*** | | | |
| 62125100 | Raisins | 62101100 | Apple, dried, uncooked |
| 63123000 | Grapes, raw, NS as to type | 63101000 | Apple, raw |
| 64116020 | Grape juice | 64104010 | Apple juice |
| ***Orange Juice for Lemon Juice*** | | | |
| 61204000 | Lemon juice, NS as to form | 61210000 | Orange juice, NFS |
| 61204010 | Lemon juice, freshly squeezed | 61210010 | Orange juice, freshly squeezed |
| 61204200 | Lemon juice, canned or bottled | 61210220 | Orange juice, canned, bottled or in a carton |
| 61204600 | Lemon juice, frozen | 61210620 | Orange juice, frozen (reconstituted with water) |
|  | | | |
| ***Peas for Asparagus*** | | | |
| 75100800 | Asparagus, raw | 75120000 | Peas, green, raw |
| 75202000 | Asparagus, cooked, NS as to form, NS as to fat added in cooking | 75224010 | Peas, green, cooked, NS as to form, NS as to fat added in cooking |
| 75202001 | Asparagus, cooked, from fresh, NS as to fat added in cooking | 75224010 | Peas, green, cooked, NS as to form, NS as to fat added in cooking |
| 75202010 | Asparagus, cooked, NS as to form, fat not added in cooking | 75224020 | Peas, green, cooked, NS as to form, fat not added in cooking |
| 75202011 | Asparagus, cooked, from fresh, fat not added in cooking | 75224021 | Peas, green, cooked, from fresh, fat not added in cooking |
| 75202012 | Asparagus, cooked, from frozen, fat not added in cooking | 75224022 | Peas, green, cooked, from frozen, fat not added in cooking |
| 75202013 | Asparagus, cooked, from canned, fat not added in cooking | 75224023 | Peas, green, cooked, from canned, fat not added in cooking |
| 75202020 | Asparagus, cooked, NS as to form, fat added in cooking | 75224030 | Peas, green, cooked, NS as to form, fat added in cooking |
| 75202021 | Asparagus, cooked, from fresh, fat added in cooking w/ butter, NFS (200394) | 75224031 | Peas, green, cooked, from fresh, fat added in cooking w/ butter, NFS (203283) |
| 75202021 | Asparagus, cooked, from fresh, fat added in cooking | 75224033 | Peas, green, cooked, from canned, fat added in cooking |
| 75202021 | Asparagus, cooked, from fresh, w/ vegetable oil, NFS (205482) | 75224031 | Peas, green, cooked, from fresh, w/ vegetable oil, NFS (201578) |
| 75202023 | Asparagus, cooked, from canned, fat added in cooking w/ butter, NFS (100156) | 75224033 | Peas, green, cooked, from canned, fat added in cooking w/ butter, NFS (100006) |
| 75202023 | Asparagus, cooked, from canned, fat added in cooking | 75224033 | Peas, green, cooked, from canned, fat added in cooking |
| 75401011 | Asparagus, from fresh, creamed or with cheese sauce | 75417011 | Peas, from fresh, creamed |
| 75401012 | Asparagus, from frozen, creamed or with cheese sauce | 75417011 | Peas, from fresh, creamed |
| ***Brussels Sprouts for Broccoli*** | | | |
| 72201100 | Broccoli, raw | 75103000 | Cabbage, green, raw |
| 72201200 | Broccoli, cooked, NS as to form, NS as to fat added in cooking | 75209000 | Brussels sprouts, cooked, NS as to form, NS as to fat added in cooking |
| 72201201 | Broccoli, cooked, from fresh, NS as to fat added in cooking | 75209001 | Brussels sprouts, cooked, from fresh, NS as to fat added in cooking |
| 72201202 | Broccoli, cooked, from frozen, NS as to fat added in cooking | 75209000 | Brussels sprouts, cooked, NS as to form, NS as to fat added in cooking |
| 72201210 | Broccoli, cooked, NS as to form, fat not added in cooking | 75209010 | Brussels sprouts, cooked, NS as to form, fat not added in cooking |
| 72201211 | Broccoli, cooked, from fresh, fat not added in cooking | 75209011 | Brussels sprouts, cooked, from fresh, fat not added in cooking |
| 72201212 | Broccoli, cooked, from frozen, fat not added in cooking | 75209012 | Brussels sprouts, cooked, from frozen, fat not added in cooking |
| 72201220 | Broccoli, cooked, NS as to form, fat added in cooking w/ butter, NFS (200170) | 75209021 | Brussels sprouts, cooked, from fresh, fat added in cooking w/ butter, NFS (201523) |
| 72201220 | Broccoli, cooked, NS as to form, fat added in cooking | 75209021 | Brussels sprouts, cooked, from fresh, fat added in cooking |
| 72201220 | Broccoli, cooked, NS as to form, w/ vegetable oil, NFS (201276) | 75209021 | Brussels sprouts, cooked, from fresh, w/ vegetable oil, NFS (206535) |
| 72201221 | Broccoli, cooked, from fresh, w/ animal fat or meat drippings (205270) | 75209021 | Brussels sprouts, cooked, from fresh, w/ animal fat or meat drippings (207320) |
| 72201221 | Broccoli, cooked, from fresh, fat added in cooking w/ butter, NFS (100033) | 75209021 | Brussels sprouts, cooked, from fresh, fat added in cooking w/ butter, NFS (201523) |
| 72201221 | Broccoli, cooked, from fresh, fat added in cooking | 75209021 | Brussels sprouts, cooked, from fresh, fat added in cooking |
| 72201221 | Broccoli, cooked, from fresh, fat added in cooking w/ vegetable oil, NFS (200428) | 75209021 | Brussels sprouts, cooked, from fresh, w/ vegetable oil, NFS (206535) |
| 72201222 | Broccoli, cooked, from frozen, fat added in cooking w/ butter, NFS (200439) | 75209022 | Brussels sprouts, cooked, from frozen, w/ butter, NFS (203013) |
| 72201222 | Broccoli, cooked, from frozen, fat added in cooking | 75209022 | Brussels sprouts, cooked, from frozen, fat added in cooking |
| 72201222 | Broccoli, cooked, from frozen, w/ vegetable oil, NFS (202783) | 75209022 | Brussels sprouts, cooked, from frozen, w/ vegetable oil (101313) |
| ***Cod for Shrimp*** | | | |
| 26319110 | Shrimp, cooked, NS as to cooking method | 26109110 | Cod, cooked, NS as to cooking method |
| 26319120 | Shrimp, baked or broiled w/ animal fat or drippings (206184) | 26109120 | Cod, baked or broiled w/ butter, NFS (202670) |
| 26319120 | Shrimp, baked or broiled w/ butter, NFS (200875) | 26109120 | Cod, baked or broiled w/ butter, NFS (202670) |
| 26319120 | Shrimp, baked or broiled w/o fat (202863) | 26109120 | Cod, baked or broiled w/o fat (201039) |
| 26319120 | Shrimp, baked or broiled | 26109120 | Cod, baked or broiled |
| 26319120 | Shrimp, baked or broiled w/ vegetable oil, NFS (200498) | 26109120 | Cod, baked or broiled w/ vegetable oil, NFS (201359) |
| 26319130 | Shrimp, steamed or boiled | 26109160 | Cod, steamed or poached |
| 26319140 | Shrimp, floured, breaded, or battered, fried w/ butter, NFS (206736) | 26109140 | Cod, floured or breaded, fried w/ butter, NFS (206663) |
| 26319140 | Shrimp, floured, breaded, or battered, fried | 26109140 | Cod, floured or breaded, fried |
| 26319140 | Shrimp, floured, breaded, or battered, fried w/ margarine, NFS (205530) | 26109140 | Cod, floured or breaded, fried w/ vegetable oil, NFS (204165) |
| 26319140 | Shrimp, floured, breaded, or battered, fried w/ vegetable oil, NFS (201330) | 26109140 | Cod, floured or breaded, fried w/ vegetable oil, NFS (204165) |
| 26319180 | Shrimp, canned | 26109110 | Cod, cooked, NS as to cooking method |

^1^Six-digit numbers in parentheses denote food modification codes (if applicable). Modification codes are the way in which FNDDS further specifies a food item in order to calculate nutritional information. Often these codes clarify which fat was used in cooking, where the main food item is described simply as “fat added in cooking.”
